# Ephrin-A5 and EphA5 Interaction Induces Synaptogenesis during Early Hippocampal Development

**DOI:** 10.1371/journal.pone.0012486

**Published:** 2010-08-31

**Authors:** Yukio Akaneya, Kazuhiro Sohya, Akihiko Kitamura, Fumitaka Kimura, Chris Washburn, Renping Zhou, Ipe Ninan, Tadaharu Tsumoto, Edward B. Ziff

**Affiliations:** 1 Department of Biochemistry, New York University School of Medicine, New York, New York, United States of America; 2 Division of Neurophysiology, Osaka University Graduate School of Medicine, Suita, Japan; 3 Brain Science Institute, RIKEN, Wako, Japan; 4 Laboratory for Cancer Research, College of Pharmacy, Rutgers University, Piscataway, New Jersey, United States of America; 5 Department of Psychiatry, New York University School of Medicine, New York, New York, United States of America; INSERM U901, France

## Abstract

**Background:**

Synaptogenesis is a fundamental step in neuronal development. For spiny glutamatergic synapses in hippocampus and cortex, synaptogenesis involves adhesion of pre and postsynaptic membranes, delivery and anchorage of pre and postsynaptic structures including scaffolds such as PSD-95 and NMDA and AMPA receptors, which are glutamate-gated ion channels, as well as the morphological maturation of spines. Although electrical activity-dependent mechanisms are established regulators of these processes, the mechanisms that function during early development, prior to the onset of electrical activity, are unclear. The Eph receptors and ephrins provide cell contact-dependent pathways that regulate axonal and dendritic development. Members of the ephrin-A family are glycosyl-phosphatidylinositol-anchored to the cell surface and activate EphA receptors, which are receptor tyrosine kinases.

**Methodology/Principal Findings:**

Here we show that ephrin-A5 interaction with the EphA5 receptor following neuron-neuron contact during early development of hippocampus induces a complex program of synaptogenic events, including expression of functional synaptic NMDA receptor-PSD-95 complexes plus morphological spine maturation and the emergence of electrical activity. The program depends upon voltage-sensitive calcium channel Ca^2+^ fluxes that activate PKA, CaMKII and PI3 kinase, leading to CREB phosphorylation and a synaptogenic program of gene expression. AMPA receptor subunits, their scaffolds and electrical activity are not induced. Strikingly, in contrast to wild type, stimulation of hippocampal slices from P6 EphA5 receptor functional knockout mice yielded no NMDA receptor currents.

**Conclusions/Significance:**

These studies suggest that ephrin-A5 and EphA5 signals play a necessary, activity-independent role in the initiation of the early phases of synaptogenesis. The coordinated expression of the NMDAR and PSD-95 induced by eprhin-A5 interaction with EphA5 receptors may be the developmental switch that induces expression of AMPAR and their interacting proteins and the transition to activity-dependent synaptic regulation.

## Introduction

Glutamatergic synapses of the CNS are highly dynamic excitatory structures that form and mature through a complex series of steps, including the adhesion of dendritic filopodia to axonal membranes, the formation of the cytoarchitecture that governs spine morphology, and the maturation of spines from an elongated to a compact mushroom shape. One step that is critical for synapse function is the recruitment of glutamate receptors to the synaptic membrane, with N-methyl-D-aspartate receptors (NMDARs) recruited initially, and (±)-α-amino-3-hydroxy-5-methyl-4-isoxazolepropionic acid receptors (AMPARs) recruited later by activity-dependent mechanisms involving the NMDA receptor [1]. Each step may be regulated by neuronal activity, which is identified specifically as the activity arising from electrical signals induced in circuits of neurons via synapses.

Mature glutamatergic synapses express both NMDAR and AMPAR [2]. NMDARs are comprised of NR1, NR2A-D and NR3A and 3B subunits, whereas the AMPARs are assembled from subunits GluR1-4. These subunits make specific interactions with scaffolding proteins. NR2A and NR2B may be bound to the postsynaptic density protein, PSD-95, while GluR2 and GluR3 form complexes with two related scaffolds, glutamate receptor interacting protein (GRIP) [3] and AMPAR binding protein (ABP) [4]. The mechanism that coordinates the expression of these proteins during early neuronal development remains largely unexplained.

Early neuronal development presents a novel problem for synapse genesis and activation. NMDAR activity, which contributes extensively to synapse plasticity including AMPAR trafficking, requires membrane depolarization to relieve the NMDAR Mg^2+^ block [5], [6], [7]. Because AMPAR conduct the major currents that provide this depolarization, excitatory synapses that lack AMPARs are functionally immature (silent). In early development, such functionally immature synapses are abundant and the level of electrical activity is low, and hence mechanisms of neuronal maturation that depend on electrical activity and the NMDAR are not yet operative. Such a situation arises during the development of hippocampus, cerebral cortex and cerebellum, regions where immediately following migration of neurons from the ventricular zone of the neural tube, neurons distribute into laminar formations [8], [9]. At this developmental stage, conventional electrophysiological activity is limited or non-existent. This suggests that activity-independent signaling pathways may guide initial synapse maturation during early development.

Signals transmitted via cell-cell contact provide an alternative means for regulating early stage synaptogenesis. Cell-cell contacts are extensive in the developing hippocampus, and are initiated when dendritic filopodia make contact with axons and are stabilized by adhesion of pre- and postsynaptic membranes. One group of contact-dependent pathways utilizes adhesive receptors and their ligands, including the Eph receptors and ephrins. EphA or EphB family receptors are activated generally by ephrin-A or ephrin-B ligands, respectively [10], [11]. Members of the ephrin-A family are glycosyl-phosphatidylinositol-anchored to the cell surface, whereas the ephrin-B family are transmembrane proteins [12]. EphB receptor signals play important roles in axonal pathfinding, establishment of topographic projections during development [13], morphological alteration of filopodia to mature-shaped spines, and clustering with the NMDAR [14]. In the adult, EphB receptors are implicated in forms of synaptic plasticity [15] and modulate NMDAR-mediated Ca^2+^ influx and gene expression [16]. It has been shown that multiple EphB receptors contribute directly to spine formation [17], [18] through activation of focal adhesion kinase [19], [20] and Rho family GTPases and their GEFs [21], [22]. In contrast, the established functions of the EphA receptor family are mainly restricted to topographic mapping of neurons in the brain [23], although ephrinA3/EphA4, the latter of which is found in spines of adult [24], contribute to hippocampal spine maturation [25] and spine collapse [26]. Notably, the role of ephrin/Eph signaling in synaptogenesis during the early stages of development has not been established.

Here we examine the morphological and functional effects of ephrinA5-EphA5 receptor signaling and the pathways that transduce these effects. We show that signaling that is induced by ephrin-A5-EphA5 receptor interaction contributes to early stage synaptogenesis. The interaction of ephrin-A5 with EphA5 leads to the activation of voltage sensitive Ca^2+^ channels (VSCCs), which in turn elevates cAMP levels, leading to a PKA-dependent induction of the expression of NMDAR subunits and PSD-95, potentially via the CREB transcription factor, which is also activated. We show that the cAMP/PKA pathway stimulates PI3 kinase to activate cdc42 and induce filopodia. These filopodia mature into mushroom shaped spines through VSCC induction of CaMKII. Notably, in hippocampal slices from P5 EphA5 receptor functional knockout mice, this program does not operate and the NMDAR currents characteristic of the wild type are not detected. This establishes ephrin-A5-EphA5 interaction as an electrical activity-independent initiator of signals that stimulate early stage synaptogenesis that may be facilitated by pyramidal cell-cell contacts found in hippocampal laminar assemblies.

## Results

### Expression and interaction of ephrin-A5 and EphA5

**Figure 1:**
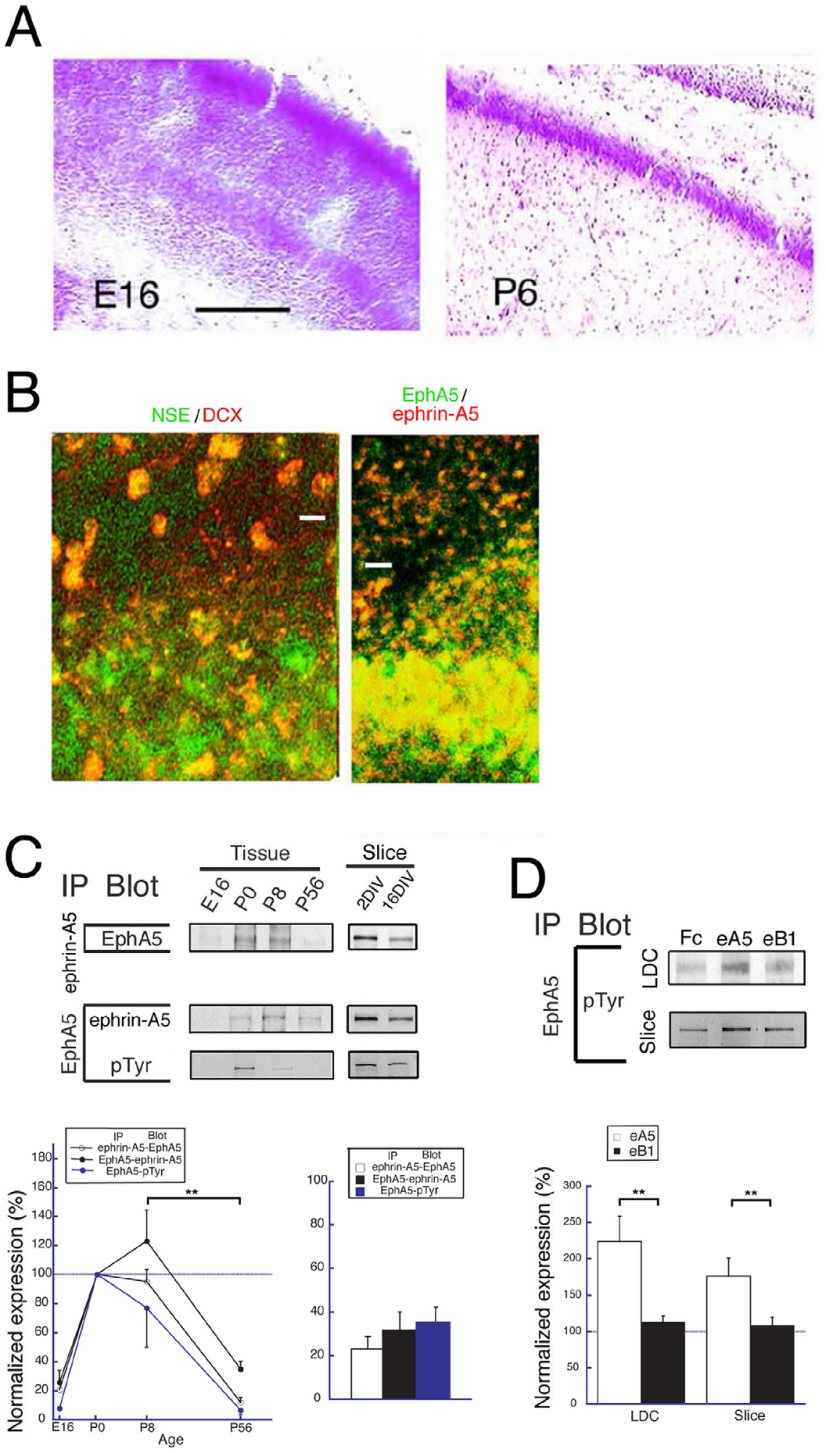
Interaction of EphA5 and ephrin-A5. (A) Nissl staining of the CA1 region of the hippocampus. The laminar organization increases from E16 to P6. Bar; 100 mm. (B) Immunostaining for hippocampal tissues at P4. Colors of letters indicate the antibodies used in this and subsequent figures. (C) IP was performed with hippocampal tissues and slice cultures of the indicated age. pTyr; phospho-tyrosine. n = 5 and 4, for tissues and slices, respectively. (D) IP was performed with hippocampal low-density dissociated cell cultures (LDC) and slice cultures treated with eA5 or eB1. n = 4, all for LDC and slices. ** indicate significance at P less than 0.05.

To begin to determine the role of ephrin-Eph receptor signaling in synaptogenesis in early development, we assessed both the expression in hippocampal tissue at the embryonic and postnatal stages of ephrins and Eph receptors and the contacts between cells necessary for ephrin-Eph receptor interaction and signaling. Nissl staining revealed that neurons in the CA1 region are sparsely distributed at embryonic day 16 (E16), whereas they are closely associated and in contact at postnatal day 6 (P6) (Figure 1A). Moreover, immunostaining of slice cultures at 4 days *in vitro* (DIV) (slice cultures were started at P0) with antibodies to neuron specific enolase (NSE) and doublecortin (DCX), which identify mature and immature neurons respectively, showed that mature neurons are closely associated in the laminar formation while immature neurons are sparsely distributed in the stratum radium in the CA1 region (Figure 1B, left panel). Significantly, the closely associated neurons of the laminar formation were immunostained with both anti-ephrin-A5 and EphA5 antibodies, while the sparse neurons of the stratum radium were not well stained (Figure 1B; right panel). These results suggest that mature neurons in the hippocampal laminar formation form extensive neuron-neuron contacts and express both ephrin-A5 and EphA5 at the early stage of development (4 DIV).

To determine whether ephrin-A5 and EphA5 interact functionally in hippocampus and if the interactions are regulated developmentally, we immunoprecipitated (IP'ed) these receptors from hippocampal tissues and from slice cultures at different stages of development and on different culture days. In tissues, complexes of ephrin-A5 with EphA5 were prominent at the neonatal period (P0, P8), but were not detected or were lower at the embryonic (E16) and later (P56) developmental stages (Figure 1C; left upper and middle panels, and graph on left). In slice cultures, the interaction was significantly decreased at 16 days *in vitro* (DIV) in comparison to 2 DIV (Figure 1C; right upper and middle panels, and graph on right). Moreover, both in the tissues and the slice cultures, the levels of endogenous phosphorylation of EphA5 paralleled the interaction of ephrin-A5 with EphA5 (Figure 1C; bottom panels, and graphs). These results suggested that the interaction of ephrin-A5 with EphA5 detected by IP predominated at the early postnatal developmental stages and was functional.

To determine whether activation of EphA5 by extracellular ligand induces EphA5 phosphorylation in these neurons, we stimulated neurons by adding a chimeric protein in which an ephrin extracellular domain is fused to the IgG C-terminal Fc region. These chimeras were ephrin-A5/Fc (eA5; 200 ng/ml) or ephrin-B1/Fc (eB1; 200 ng/ml) and had been clustered with IgG antibody (Fc). The chimeras were added to developing low-density dissociated neuron cultures and slice cultures for the period during which ephrin-A5-EphA5 interaction was observed *in vivo*. We reasoned that in these low-density culture conditions or in slices, the cell-cell contacts that lead to ephrin-A5-EphA5 interaction would be diminished and the activation of EphA5 would reflect the action of exogenous chimeric ligand. Thus, to mimic the *in vivo* period of stimulation by ephrin-A5, we added ligand to low-density dissociated cultures at embryonic day 18 (E18) for 6 days and to slice cultures at P0 for 4 days. Significantly, under these conditions, eA5, but not the controls, Fc or eB1, induced phosphorylation of EphA5 (Figure 1D). Furthermore, under neither culture condition did the level of EphA5 change with either eA5 or eB1 treatment. Also, the expression of ephrin-A5 in hippocampal tissues was not changed in EphA5-functional knockout mice at P5-6 (EphA5^lacZ/lacZ^) relative to wild type (Figure S1) ruling out receptor expression changes as the basis for the increased EphA5 phosphorylation. We conclude that exogenous ephrin-A5 ligand may lead to ligand specific endogenous EphA5 receptor activation, as reflected by receptor phosphorylation.

### Failure of NMDAR neurotransmission in EphA5-functional knockout mice

**Figure 2:**
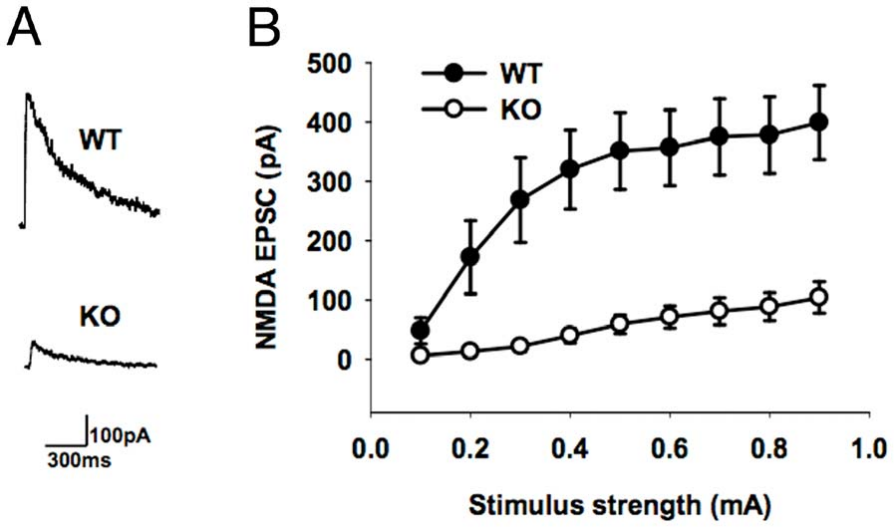
Absence of evoked NMDA receptor currents in hippocampus during early development of EphA5 functional knockout mice. (A) Examples of NMDA EPSCs evoked in the CA1 pyramidal neurons in hippocampal slices from postnatal day 6 EphA5-functional knockout and matched wild-type mice. (B) Average amplitude of NMDA EPSCs in EphA5-functional knockout (15 slices from 5 mice) and matched wild-type mice (15 slices from 5 mice). EphA5-functional knockout mice show significantly lower NMDA EPSC amplitude compared to the matched wild-type mice. P<0.001, two-way ANOVA.

An early step in the acquisition of glutamatergic synapse function is the expression of synaptic NMDA receptors [1]. If the early phases of hippocampal synaptogenesis depend on EphA5, the onset of early hippocampal electrical activity as manifested by NMDAR currents should depend on EphA5 receptor signaling. In this case, early electrical activity and NMDAR function should be absent or greatly decreased in EphA5 receptor functional knockout mice. To determine whether NMDA receptor neurotransmission is affected in the EphA5-functional knockout mice, we studied the amplitude of NMDA EPSCs in CA1 pyramidal neurons in hippocampal slices from EphA5-functional knockout mice and matched wild-type mice on postnatal day 6. EphA5-functional knockout mice showed significantly lower NMDA EPSC amplitude compared to their wild-type littermates (Figure 2A, B; p<0.001, Two-way ANOVA). These results support the conclusion that EphA5 signaling is necessary for normal development of NMDA receptor neurotransmission and the onset of electrical activity.

### The EphA5 signal regulates cAMP via VSCC

**Figure 3:**
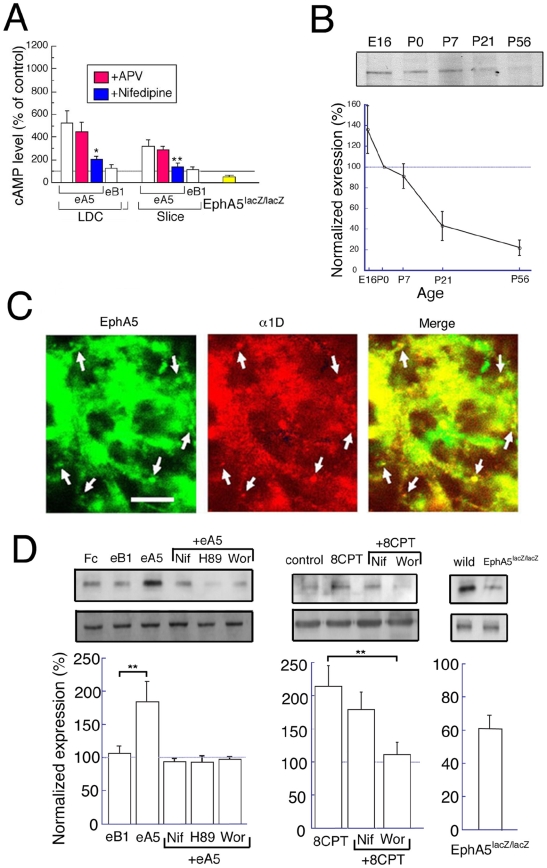
Intracellular signal transduction by EphA5. (A) The intracellular level of cAMP was measured for LDC, for slice culture with or without eA5 or eB1 with or without APV (50 µM) or nifedipine (20 µM)(white, red, blue bars) and for EphA5 ^lacZ/lacZ^ at P4 (yellow bar). The data from EphA5 ^lacZ/lacZ^ were normalized to wild-type, whereas the other data were normalized to control without drug. n = 4, all for LDC, slices and *in vivo*. (B) Western blot analysis was performed for the developmental expression of α1D in hippocampal tissues at the indicated ages (upper panel). n = 4. (C) Double immunostaining with anti-EphA5 and anti-α1D antibodies was performed for hippocampal neurons in culture for 4 DIV (upper panel). Arrows indicate representative colocalization of EphA5 and α1D in puncta in proximal neurites. Bar indicates 10 µm. (D) For measurement of cdc42 activity, hippocampal neurons were cultured for 6 DIV with or without the indicated drug(s), and hippocampal tissues from EphA5^lacZ/lacZ^ and wild-type mice at P5-6 were dissected. Then, activated and total cdc42 were measured as shown in Methods. Upper and lower panels indicate representative blots of activated cdc42 and total cdc42, respectively. Activated cdc42 values are normalized to the level of total cdc42. n = 5–6 and 4, for LDC and *in vivo*, respectively. ** indicate significance at P less than 0.05.

The mechanisms by which EphA5 may contribute to synaptogenesis have not been extensively investigated. However, because cAMP and the cAMP-dependent protein kinase, PKA, have central roles in neuronal signaling [27], and because EphA5 induces filopodia in non-neuronal cells by a mechanism involving cAMP [28], we investigated the control of cAMP levels by EphA5 signal transduction. When we repeated the activation of EphA by eA5 in low-density culture neurons or in slice cultures, the eA5 chimera increased the levels of intracellular cAMP. Also, the level of endogenous cAMP was significantly lower in hippocampal tissues from EphA5^lacZ/lacZ^ mice relative to the wild-type (Figure 3A). These results suggested that stimulation of EphA receptors elevated cAMP levels.

We next investigated the mechanism of elevation of cAMP. In mature neurons, activity dependent Ca^2+^ fluxes activate Ca^2+^ - regulated adenylate cyclases, which produce cAMP [29]. Voltage-sensitive calcium channels (VSCCs) and NMDARs are the main Ca^2+^ channels in neurons [30]. Significantly, nifedipine (20 µM) (VSCC inhibitor), but not (D)-2-amino-5-phosphonovaleric acid (50 µM) (APV, NMDAR antagonist) blocked the increase in cAMP following neuron treatment with eA5 (Figure 3A). These results suggest that the EphA5 signaling pathway that increases cAMP depends upon the activation of VSCCs, but not NMDARs. Indeed, expression of VSCCs as reflected by the presence of their α1D subunit, was observed from the embryonic to postnatal stages in hippocampal tissues, paralleling the expression pattern of EphA5, with the expression gradually declining in the adult (Figure 3B) [31], [32], [33]. Also, EphA5 colocalized with α1D in primary hippocampal neuron cultures (Figure 3C), supporting the possibility that VSCCs containing α1D were activated by EphA5 signaling. These results suggest that during early hippocampal development, the elevation of cAMP by EphA5 signaling depends upon VSCC activation.

### EphA5 controls Cdc42 via PKA and PI3K

The next effect of cAMP that we examined was the induction of Cdc42, which lies on pathways that lead to spine formation. Cdc42 contributes to the formation of filopodia, which are among the initial morphological structures that can lead to spine formation. Cdc42 is regulated by phosphatidylinositol-3-kinase (PI3K) [6]. Indeed, EphA5 and cAMP induce filopodia in non-neuronal cells via Cdc42 and PI3K [28], and PKA can control PI3K [34]. This suggested that EphA5 activation of PKA may activate PI3K and cdc42. In this case, an EphA5, VSCC, cAMP, PKA, PI3K, cdc42 pathway may induce filopodia. To determine whether the activation of cdc42 by PI3K is one step in such a pathway, we asked whether VSCCs, PKA and PI3K induced by EphA5 stimulation control Cdc42 activity. Indeed, treatment with eA5, but not eB1, significantly enhanced the activation of Cdc42, and this activation was completely blocked by nifedipine, by _H89_ (10 µM) (PKA inhibitor) and by wortmannin (100 nM) (PI3K inhibitor) (Figure 3D, left panels and graph). These results suggest that VSCC, PKA and PI3K signals are downstream of the EphA signal and can activate cdc42. In support, the level of activated Cdc42 was significantly reduced in tissues from EphA5^lacZ/lacZ^ hippocampus in comparison to wild-type (Figure 3D, right panels and graph). Also in agreement, treatment with 8CPT (1 mM) (PKA activator) enhanced the level of activated Cdc42, and this increase was almost completely blocked by wortmannin, but not by nifedipine (Figure 3D, middle panels and graph). Together, these results suggest that in hippocampal neurons, EphA5 signaling is transduced to VSCC, PKA, PI3K and Cdc42, in this order.

### EphA5 signals induce morphological changes of spines

**Figure 4:**
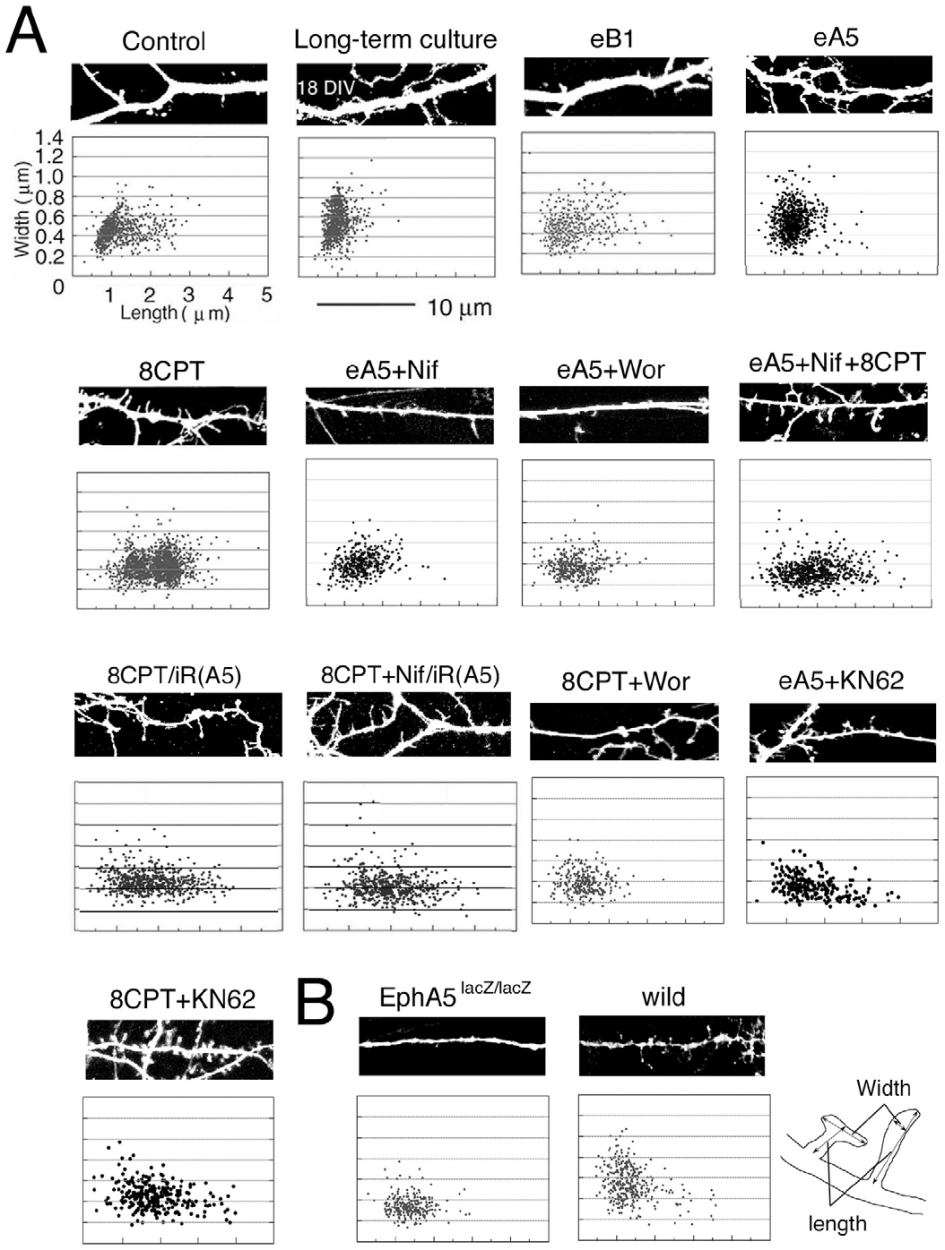
Effect of EphA5 signaling on morphology of spines. (A) Morphological analysis of spines. Hippocampal neurons were cultured for 6 DIV or 17–20 DIV (long term culture) with or without the indicated drugs). For RNAi experiments, pGFP was transfected with or without the siRNA (iR(A5)) into the neurons, with or without the indicated drug(s). Spine morphology width and length were analyzed in living neurons as shown in the scheme. (B) For the knockout study, the brains of EphA5^lacZ/lacZ^ and wild-type mice at P5-6 were fixed with paraformaldehyde, DiI was injected into the CA1 hippocampal neurons, and spine morphology was analyzed. Upper panels show representative images.

We next asked how PKA activity that may result from EphA5-induced increase in cAMP levels could regulate synapse function and structure. This regulation would involve both morphological changes, such as the induction of spines, and functional changes, such as the induction of glutamate receptor expression. We first analyzed the control of spine morphogenesis by the EphA5 pathway. To visualize spines, neurons in LDC were transfected with a plasmid expressing GFP (pGFP). Spines that are immature and still at the filopodial stage are tall and thin, while mature spines are predominantly short and wide (mushroom-shaped). At 6 DIV, spines in control LDC neurons showed both filopodial and mature morphologies (Figure 4A, control), whereas the in long-term culture (2–3 weeks) neurons, the majority were mature (Figure 4A, Long-term culture), as expected. eA5, but not eB1 treatment of 6 DIV LDC neurons, induced shorter and wider, more mature spines (Figure 4A, eB1 and eA5). We next analyzed the role of cAMP, which is induced by EphA5. We observed that treatment of neurons 6 DIV in LDC with the cAMP analogue, 8CPT, yielded spines that notably had a predominantly filopodial rather than mature morphology (Figure 4A, 8CPT). Thus, stimulation of PKA, although capable of inducing filopodia, was incapable of inducing maturation of spines in LDC neurons. Nifedipine completely blocked the eA5 induction of mature spines (Figure 4A, eA5+Nif), in agreement with the requirement for EphA5 activation of VSCC for cAMP production. Significantly, in the presence of nifedipine, maturation recovered upon the co-addition of 8CPT (Figure 4, eA5+Nif+8CPT), again consistent with EphA5 elevating cAMP downstream of activation of VSCC. Notably, the PI3 kinase inhibitor, wortmannin, blocked the maturation effects of both eA5 and 8CPT (Figure 4A, eA5+Wor and 8CPT+Wor), as expected if maturation depends on PKA activation of PI3 kinase, the pathway shown above to activate Cdc42. Interestingly, when neurons were treated with eA5, inclusion of KN62, a Ca^2+^/calmodulin-dependent protein kinase II (CaMKII; 2 µM) inhibitor, blocked full spine maturation, and instead filopodia were observed (Figure 4A, eA5+KN62). Moreover, KN62 did not alter the ability of 8CPT to induce filopodia (Figure 4A, 8CPT+KN62). These results suggest that following EphA5 activation and the consequent stimulation of VSCC and increase of cAMP, PKA stimulates PI3K, which activates Cdc42 and leads to the induction of filopodia, while a second signal dependent upon the activation of CaMKII induces the maturation of filopodia to mushroom shaped spines.

### EphA5 selectively induces synaptogenesis

We used RNAi knockdown to investigate the roles of individual Eph receptors in the synaptogenic pathway. To validate the knockdown procedure, we cotransfected LDC neurons with pGFP together with purified siRNAs for EphA5 (iR(A5)) or EphB1 (iR(B1)) or EphB2 (iR(B2)) or as controls, the scrambled siRNAs, iR(A5′), iR(B1′) and iR(B2′). The siRNAs iR(A5), iR(B1) and iR(B2) blocked the expression of EphA5, EphB1 and EphB2, with 65.9, 77.0 or 58.9% knockdown respectively. Each control siRNA, iR(A5′), iR(B1′) or iR(B2′) had no significant knockdown effect (Figure S2A and B). Similar to the result with the EphA5 scrambled siRNA control, iR(A5′), neither the heterologous experimental RNAi's, iR(B1) and iR(B2), nor the heterologous control RNAi's, iR(B1′) and iR(B2′) showed significant knockdown of EphA5 (Figure S2C), indicating high specificity of the inhibitory RNAs for their targets.

In LDC neurons, the introduction of iR(A5) to knock down EphA5 did not block the ability of 8CPT or of 8CPT plus nifedipine to induce morphological changes in spines ((8CPT/iR(A5) and 8CPT+Nif/iR(A5), respectively; Figure 4A). This is in agreement with the proposed pathway, in which cAMP acts downstream from EphA5 and VSCCs. Moreover, spines of the EphA5lacZ/lacZ functional EphA5 knockout were greatly reduced in width and length compared to wild type (Figure 4B) and were similar to those of “eA5+Nif”, “eA5+Wor”, and “8CPT+Wor” neurons, in which signaling downstream from EphA5 or cAMP is blocked (Figure 4A). Together, these results suggest that specific signals from EphA5 that activate PKA and PI3K via VSCC, and that activate CaMKII, result in spine formation and maturation, respectively.

### EphA5 signals induce expression of NMDA receptor subunits

**Figure 5:**
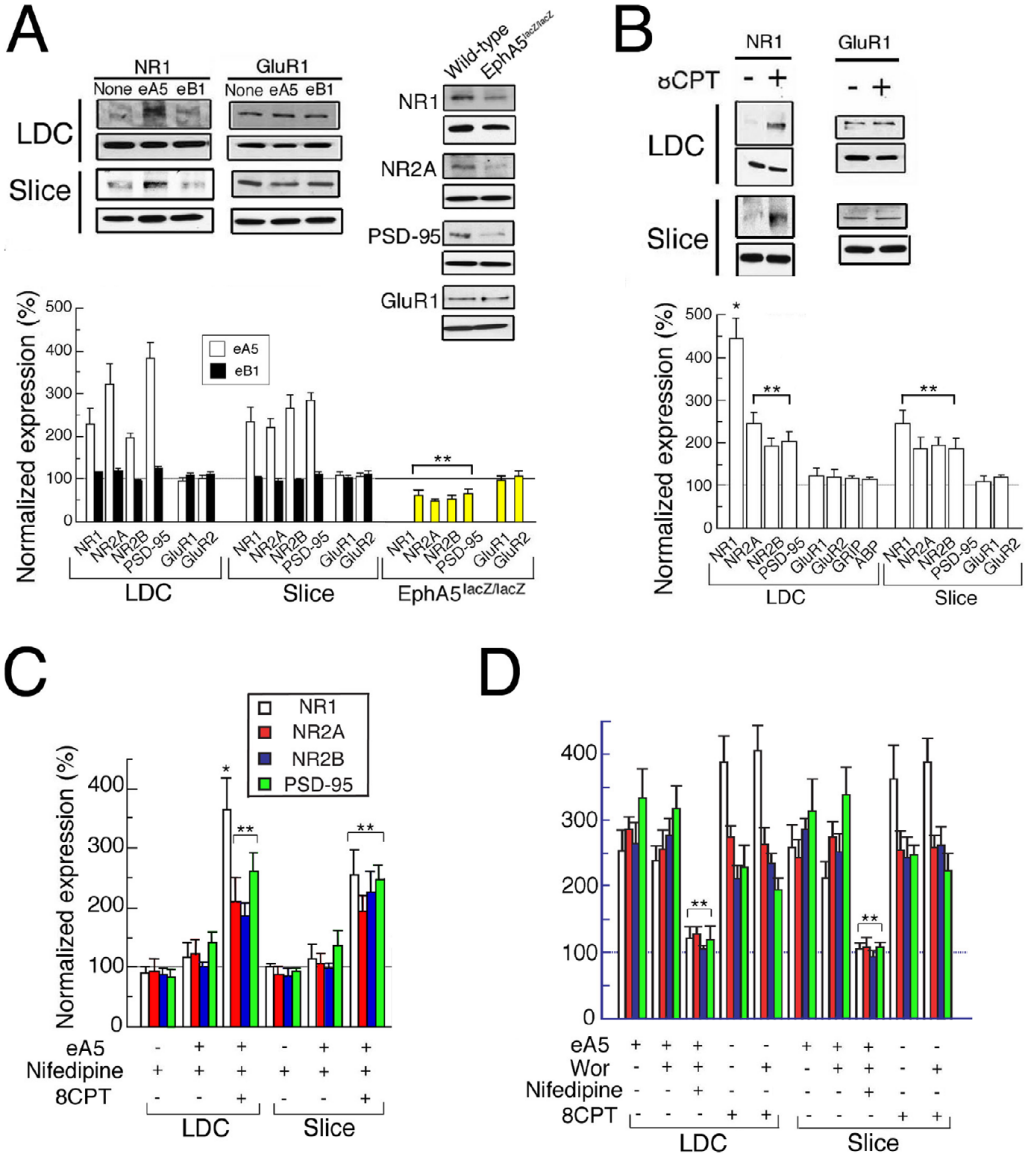
Regulation of expression of glutamate receptors by EphA5 signaling. (A) Western blotting of glutamate receptor subunits and PSD-95 from LDC at 6 DIV or slice cultures at 4 DIV without (control), or with eA5 or eB1 and EphA5^lacZ/lacZ^ at P 4. n = 5–7, 4–5 and 4, for LDC, slices and *in vivo*, respectively. Tubulin loading control blots are shown. (B) Western blotting of glutamate receptor subunits, PSD-95, GRIP1 and ABP (GRIP2) from LDC at 6 DIV or slice cultures at 4 DIV without (control) or with 8CPT (1 mM). n = 5–6, and 4, for LDC and slices, respectively. (C, D) Western blotting of glutamate receptor subunits and PSD-95 from LDC at 6 DIV or slice cultures at 4 DIV without (control) or with the indicated drug(s). n = 4–6 and 4–5, for LDC and slices, respectively. * and ** indicate p<0.01 and p<0.05, respectively, by ANOVA versus without the drug(s).

The expression of glutamate receptors is an essential step in the program of glutamatergic synapse formation. Amongst the glutamate receptors, the expression of NMDARs precedes that of AMPARs developmentally [35] and thus NMDARs are a marker for an early development function that may be induced by ephrin-A5-EphA5. Significantly, neurons that had been grown in low density culture (LDC) and neurons in slice cultures, when treated with eA5 but not with eB1, increased expression of the NMDAR subunits NR1, NR2A and NR2B as well as the expression of PSD-95 [36] as detected by Western blotting (Figure 5A). The levels of α-tubulin, a control, remained the same. In contrast to the induction of the NMDAR subunits, expression of the AMPAR subunits GluR1 and GluR2 was not significantly changed (Figure 5B). Moreαover, in EphA5^lacZ/lacZ^ hippocampal tissues, expression of NMDAR subunits and PSD-95, but not of GluR1 or GluR2, was significantly reduced compared to wild type (Figure 5B). This suggested that EphA5 activity specifically induces NMDAR expression.

To determine whether cAMP, such as is elevated by EphA5, was sufficient to induce NMDAR subunit expression, we treated LDC neurons or slice cultures with 8CPT for 6 DIV. As seen with activation of EphA5, 8CPT increased expression of NMDAR subunits and PSD-95, but the levels of neither GluR1 nor GluR2 were chan ged (Figure 5B). We also found that nifedipine almost completely blocked the EphA5, but not the 8CPT activation of NMDAR subunits and PSD-95 (Figure 5C). This was as expected if the induction of cAMP is downstream from the activation of VSCCs, and supported the proposal that EphA5 induces Ca^2+^ influx via VSCCs, which elevates cAMP to stimulate NMDAR subunit and PSD-95 expression. Because wortmannin did not block the expression of NMDAR subunits or PSD-95, although nifedipine did (Figure 5D), PI3K is not involved in NMDAR subunit or PSD-95 expression. This is in contrast to the activation of cdc42, which depends on PI3K (Figure 3D). NR1 transcripts increased approximately eightfold relative to control following the treatment of LDC neurons with 8CPT (Figure S3), suggesting that the regulation by PKA was transcriptional.

### Eph signaling induces NMDAR subunit complexes

**Figure 6:**
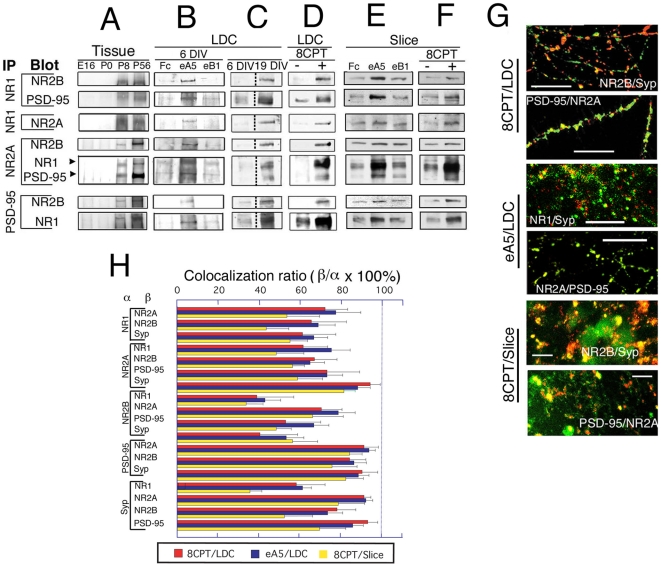
Stoichiometry and co-localization of NMDAR complex induced by EphA5 signaling. IP was performed (A) for hippocampal tissues of the indicated ages. (B–D) for neurons cultured for 6 DIV (B–D) or 19 DIV (C) or for slice culture for 4 DIV (E, F) in the following conditions: with Fc, eA5 or eB1 (B, E), at LDC, 18×10^3^ cells/cm^2^) (C), without or with 8CPT (D, F). (G, H) Similar effects of 8CPT and eA5 on distribution of NMDAR subunit proteins and PSD-95 at synapses. (C) indicates the same membrane, but the signals do not border on. (G) Examples of immunocytochemical analyses of effects of 8CPT or eA5 on LDC and slice culture. Neurons cultured in LDC and slice culture with 8CPT or eA5 for 6 and 4 DIV, respectively, were stained with the indicated antibodies (green/red). Bar indicates 10 µm. Syp: synaptophysin. (H) Colocalization ratio of NMDAR subunits, PSD-95 and synaptophysin in neurons cultured with 8CPT or eA5. Neurons cultured in LDC and in slice culture with 8CPT or eA5 for 6 and 4 DIV, respectively, were stained with the indicated combinations of antibodies (α versus β), and ratios were calculated as: number of yellow dots on merged image (β)/number of all the dots on single color image of the interest protein (α)}×100%. The extents of colocalization were similar (P>0.05) for the three culture conditions, supporting the similarity of the eA5 and 8CPT induced complexes.

We next determined by IP and Western blot analysis whether the NMDAR subunits and PSD-95 induced by EphA5 bound to one another. We detected NR1/NR2A/NR2B/PSD-95 complexes *in vivo* in hippocampus, and the levels of these complexes increased developmentally from E16 to adult (Figure 6A). In LDC neurons and slice cultures, eA5, but not eB1, enhanced the expression of each NMDAR subunit and of PSD-95 (Figures 6B and 6E). Interestingly, the subunit compositions of complexes from older animals (P8) resembled complexes from older LDC neurons (19 DIV) but not younger ones (6 DIV) (Figure 6C). Also, 8CPT treatment of LDC neurons (6 DIV) or slice culture neurons induced NMDAR complexes that resembled those of eA5 treated cells (NR1/NR2A/NR2B/PSD-95 complexes) (Figures 6D and 6F), again consistent with cAMP regulating the expression of these proteins, under EphA5 control. We conclude that EphA5 and cAMP induce NMDAR complexes that are characteristic of mature neurons. Notably, the NMDAR subunits and PSD-95 that were induced by eA5 or 8CPT colocalized with synaptophysin, a synapse marker (Figure 6G, H). These immunocytochemical results further suggest eA5/EphA5 signaling and its downstream second messenger, cAMP, induce NMDAR complexes that are at synapses and are functional.

### Electrical activity is induced by Eph signaling

**Figure 7:**
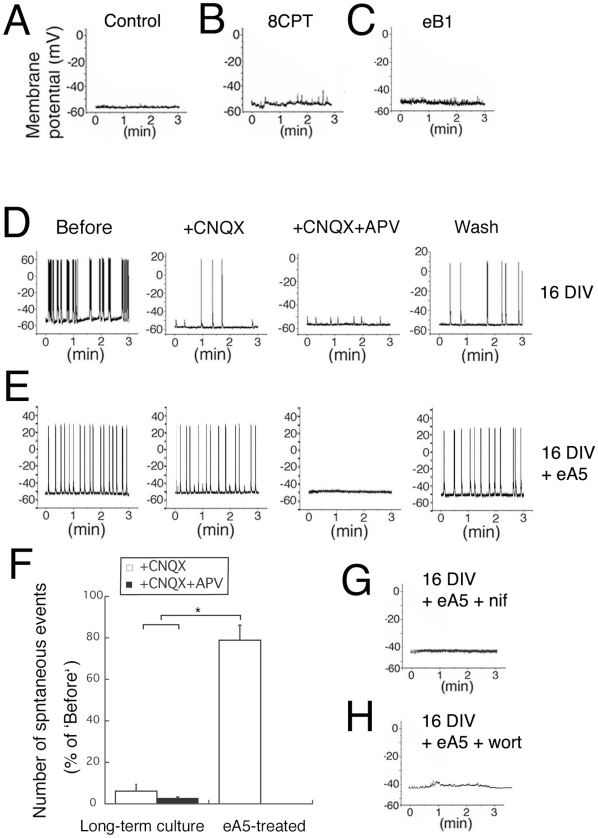
EphA5 signals induce electrical activity. The current-clamp technique was employed with neurons cultured under the following conditions: (A) control, 6 DIV, (B) with 8CPT, 6 DIV, (C) with eB1, 6 DIV, (D) without drug, 16 DIV, (E) with eA5, 6DIV, (F) with eA5 and nifedipine, 6DIV, (G) eA5 and wortmannin, 6 DIV. To ascertain that electrophysiological recordings were properly performed, after experiments 100 pA current was applied to the neuron for 500 msec (data not shown). In the control (A), or with treatment with 8CPT (B) or eB1 (C), no spontaneous responses appear. In contrast, prominent spontaneous responses were recorded in (D), long-term cultures (16 DIV) that were mostly sensitive to CNQX and completely sensitive to CNQX+APV and restored by Wash, and in (E), 6 DIV following treatment with eA5 (‘Before’). In (E) eA5-treated cultures 6 DIV, spontaneous responses were evident that were mostly insensitive to CNQX but completely sensitive to CNQX+APV and were largely restored by Wash. (F) The summarized graph of (D) and (E). The spontaneous events were counted for three minutes, and the data were normalized to the number of ‘Before’. n = 3. Spontaneous responses seen in (E) eA5-treated neurons completely disappeared with co-treatment with (G) nifedipine or (H) wortmannin. Each experiment was repeated at least two times with the same results. ** indicate significance at P less than 0.05 and * indicates significance at P less than 0.01.

To test whether the NMDAR complexes that are induced by eA5 are indeed functional, we performed direct recordings from hippocampal neurons. We did not observe spontaneous responses in the LDC control cells (Figure 7A), confirming the absence of electrical activity in 6 DIV hippocampal neurons cultured at low density. In contrast, neurons treated with eA5 in LDC showed marked spontaneous responses (Figure 7E), whereas neurons treated with 8CPT or eB1 in LDC did not show such responses (Figures 7B and 7C, respectively). This indicated that signals induced by EphA5, but not signals induced by PKA alone, could lead to the appearance of electrical activity in LDC neurons. Neurons in long-term culture (such as 16 DIV) showed marked spontaneous responses (Figures 7D; ‘Before’, panels at left). Notably, with 6 DIV eA5 treatment, the responses were from NMDAR, while responses in neurons in long-term cultures were mainly from AMPAR. This was indicated by the disappearance in long-term cultures of almost all spontaneous responses after addition of CNXQ (Figure 7D ‘+CNQX’ and 6F ‘Long-term culture, open bar’). In contrast, following treatment with 6 DIV eA5, spontaneous responses were not influenced by CNQX and were completely blocked by APV (Figures 7E ‘+CNQX’ and ‘+CNQX+APV’ and 6F ‘eA5-treated’). Notably, the ability of eA5 treatment to induce spontaneous activity was completely blocked by co-treatment with nifedipine or wortmannin (Figure 7G and 7H, respectively). These results indicate that EphA5 signaling via VSCC and PI3 kinase induces functional NMDAR complexes that are similar to those of mature neurons, and that these cells lack functional AMPAR, which in contrast are abundant in mature neurons.

### Eph signaling activates CREB

**Figure 8:**
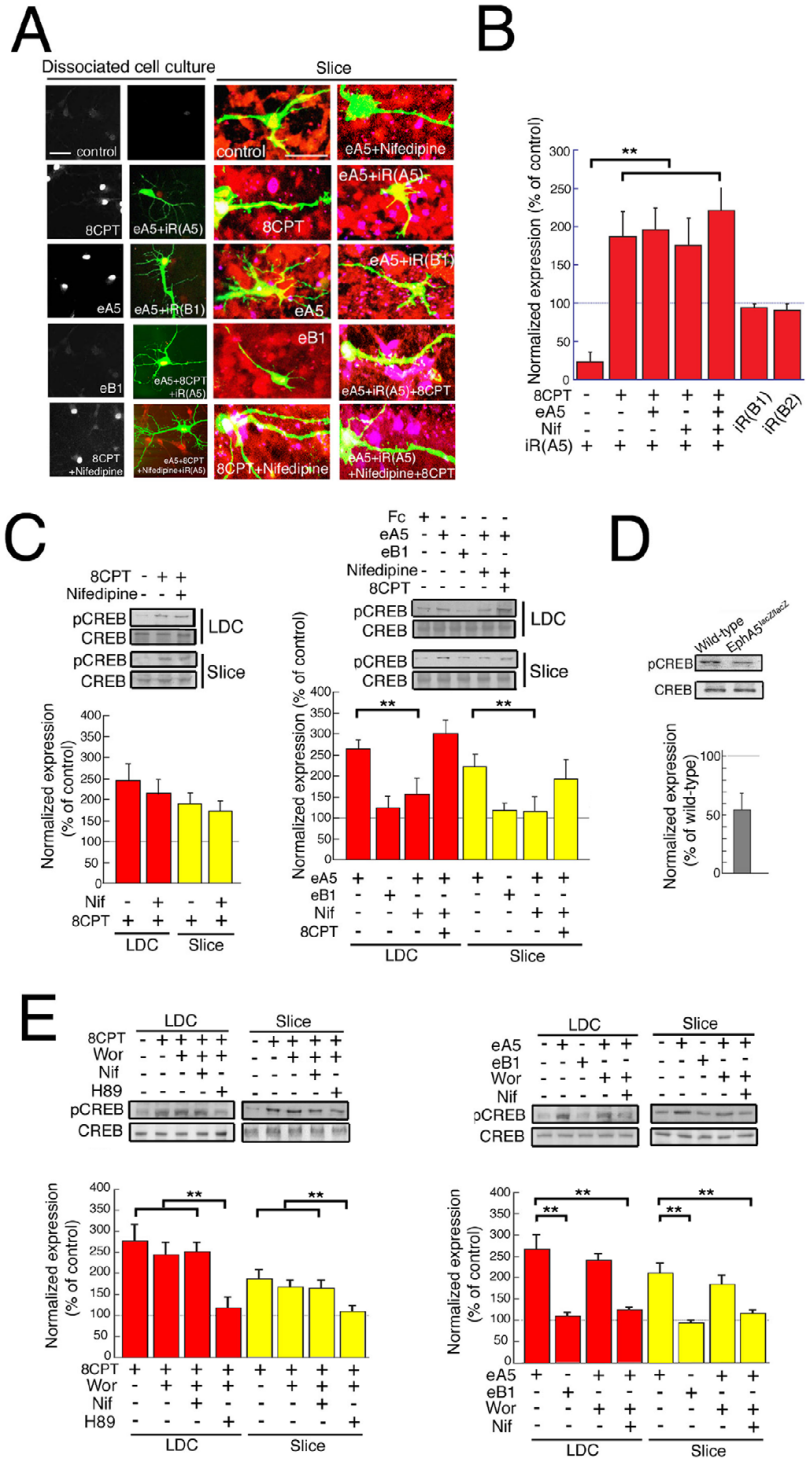
Involvement of CREB in neuronal maturation. Hippocampal neurons were cultured without or with the indicated drug(s) in LDC and in slice culture, without or with cotransfection of GFP-plasmids and the indicated siRNA. (A) Monochrome images show phospho-CREB (pCREB) staining, whereas in color images green and red indicate GFP and pCREB, respectively. Scale bar; 20 µm. For slice culture, green, red and blue indicate GFP, NSE and pCREB, respectively. (B) The graph shows the ratios (means ± SEM) of intensity of pCREB from GFP-positive and GFP-negative (neighboring) neurons in LDC. To account for experiment-experiment staining variation, we normalized all values in each condition. (C, D, E) Western blot analysis for CREB and pCREB was performed for LDC and slice culture with or without the indicated drug(s) and for EphA5 ^lacZ/lacZ^ at P4. The graphs show the ratios of pCREB to CREB expression. (D) The ratio for EphA5^lacZ/lacZ^ is normalized to that for wild-type. (B) n = 4–6, (C) n = 4–6, 4 and 4–5, for LDC and slices, respectively, (D) n = 4, (E) n = 5–7 and 4–6, for LDC and slices, respectively. ** indicate significance at P less than 0.05.

Having shown that EphA5 could selectively induce functional NMDAR and scaffolds, we next asked whether EphA5 could be link to a more general program of gene expression at the transcriptional level. The transcription factor, CREB, is activated by PKA phosphorylation and is a major regulator of genes that function in synaptogenesis (West et al., 2001). Significantly, we observed that CREB phosphorylation, detected by immune fluorescence, is elevated in nuclei of eA5-treated neurons, as well as in 8CPT treated neurons, but not in control eB1-treated neurons in either LDC or slice culture (Figure 8A). Nifedipine blocked the ability of eA5 to activate CREB phosphorylation, but not the ability of 8CPT to induce CREB phosphorylation (Figure 8A), in agreement with the proposed pathway in which EphA5 activates VSCCs, elevating cAMP, leading to PKA phosphorylation of CREB. Co-transfection of iR(A5) plus pGFP, but not of iR(B1) or iR(B2), also significantly reduced phosphorylation of CREB (Figure 8A and &B), confirming the role of EphA5. Addition of 8CPT restored CREB phosphorylation in EphA5-knocked down neurons with or without treatment with eA5 and with or without nifedipine (Figure 8A and 8B), also in agreement with the pathway. Induction was sensitive to H89 (PKA inhibitor). Western blot analyses confirmed that CREB was phosphorylated following treatment with 8CPT and with eA5, but not following treatment with the control, eB1, in LDC neurons and slice culture (Figure 8C), as expected if EphA5 and cAMP lie specifically on the pathway. As further confirmation, nifedipine blocked eA5 induction of phospho CREB as seen by Western blot, but nifedipine did not block phospho CREB induction by 8CPT (Figure 8C). In EphA5^lacZ/lacZ^ mice at P4, moreover, the phosphorylated CREB/CREB ratio was markedly decreased (57.3±7.4% of wild-type; p<0.05, unpaired t-test, n = 5 and 6 for EphA5^lacZ/lacZ^ and wild type, respectively)(Figure 8D, right graph). Significantly, nifedipine, but not wortmannin, inhibited the induction of CREB phosphorylation by eA5 (Figure 8E) consistent with VSCCs but not PI3K lying on the pathway to cAMP elevation. Notably, nifedipine did not block the induction of phospho CREB by 8CPT (Figure 8E), also in agreement with the proposed pathway. These results indicate that EphA5 activates CREB phosphorylation, dependent on VSCC and PKA, thereby providing a pathway for CREB-dependent gene expression that may lead to neural maturation.

### Relevance of cell-cell contact to EphA signals

**Figure 9:**
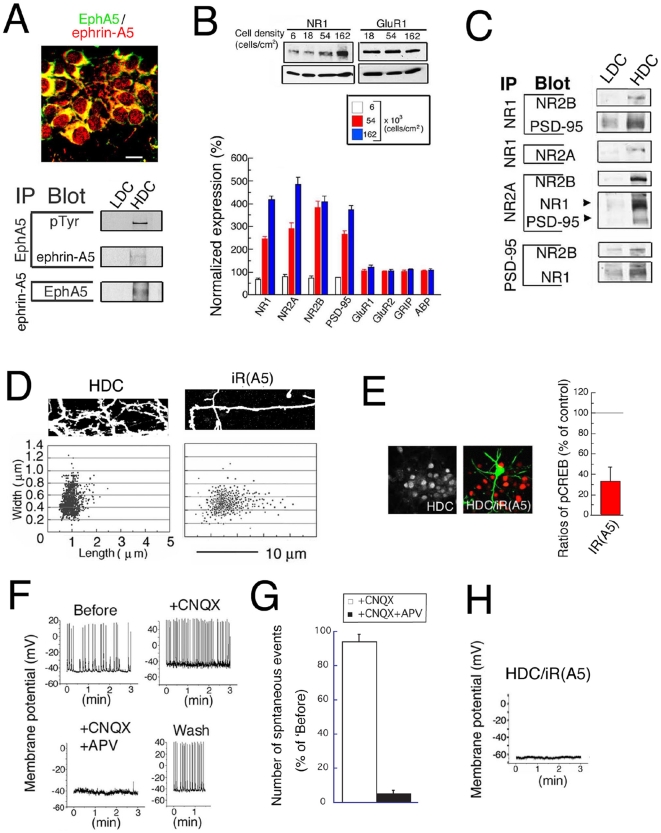
Involvement of cell-cell contact in neuronal maturation. (A) Immunostaining of neurons in HDC with ephrin-A5 (green) and EphA5 (red) (upper panel). n = 4. (B) Dissociated hippocampal neurons were incubated for 6 DIV at the indicated plating cell density (control, 18×10^3^ cells/cm^2^), and Western blot analysis was performed for NMDAR subunits, PSD-95, AMPAR subunits, GRIP1, GRIP2 and ABP. n = 5–7. (C) IP was performed with LDC and HDC as in legend of Figure 1C (lower panels). The signals from LDC are equal to those from 6 DIV in Figure 6C. (D) Hippocampal neurons were cultured in HDC for 6 DIV. After transfection of the neurons with pGFP and/or iR(A5), or iR(B1) or iR(B2), spine width and length were analyzed in living neurons as described in the legend of Figure 4. Upper panels show representative images. (E) Phosphorylation in HDC. The analysis for CREB and pCREB was performed for HDC with or without iR(A5) as the legend of Figure 8. n = 18. The graph shows the ratios (means ± SEM) of intensity of pCREB from GFP-positive and GFP-negative (neighboring) neurons in HDC. To account for experiment-experiment staining variation, we normalized all values in each condition. (F) Electrical activity in HDC. The current-clamp technique was employed with neurons cultured under the indicated conditions as in the legend of Figure 7. (G) The graph shows the summary of number of the spontaneous which were determined as described in the legend of Figure 7F. n = 3. (H) Blockade of electrical activity in the EphA5-knocked down neurons in HDC. Knockdown of EphA5 was performed as described in the legends of Figure 9D and 8E.

During the period from embryonic day 16 (E16) to postnatal day 6 (P6), neurons in hippocampus organize into lamina, in which cell bodies are arranged in close proximity to one another (Figure 1A). As lamina formation proceeds, the distances between neighboring neuronal cell bodies diminishes, thus increasing the potential for contacts of cell bodies with one another and with proximal neurites. Such contacts may in turn facilitate the function of adhesive signaling molecules, such as the ephrin-A5 and EphA5 receptors, both of which are predominantly expressed at the neonatal period (Figure 1C). Therefore, by comparing high-density culture with low-density culture, we asked whether cell-cell contact played a role in neuronal maturation induced by ephA5-EphA5 signaling. Immunostaining of neurons in HDC at 4 DIV confirmed that ephrin-A5 and EphA5 are both expressed and colocalize in these neurons (Figure 9A, upper panel). We performed IP to compare functional interactions between ephrin-A5 and EphA5 and the levels of tyrosine phosphorylation of EphA5 that may result from such interactions, in HDC where cell contact is facilitated relative to LDC. Significantly, IP revealed interaction between ephrin-A5 and EphA5, and phosphorylation of EphA5 in HDC but not in LDC neurons (Figure 9A, lower panels). Moreover, the expression of NMDAR receptor subunit subtypes and PSD-95, but neither AMPAR subunits, GluR1 and GluR2, nor the scaffolds, GRIP-1 and -2, were enhanced as the plating densities of the cultures increased (Figure 9B). IP of NMDAR subunits revealed that NMDAR complexes in HDC are of the type NR1/NR2A/NR2B/PSD-95 (Figure 9C). This was supported by the immunocytochemical results that NR1, NR2A, NR2B, PSD-95 and synaptophysin are markedly co-localized in the HDC neurons (Figure S4A). These results are also consistent with the results from eA5- and 8CPT-treated neurons in LDC (Figures 1D, 5A, 5B, 6B, 6E and 6F).

We next investigated the role of cell contact and EphA5 in establishing spine morphology. In HDC, mature spines types with wide heads and short necks were seen (Figure 9D), and these resembled spines observed in long-term cultures (Figure 4). This effect of HDC on spine morphology was blocked by EphA5 knockdown following treatment with iR(A5) (Figure 9D). In addition to EphA5, both EphB2 and EphB1 are also expressed during the embryonic to postnatal stages, and ephrinA5 can induce the phosphorylation of EphB2 as well as of EphA5 [37]. However, unlike iR(A5), neither iR(B1) nor iR(B2) affected the mature spine morphology seen in HDC (Figure 9D), confirming the specificity of the role of EphA5 in spine maturation in HDC. CREB phosphorylation was prominent in HDC (Figure 9E), and was blocked by treatment with nifedipine or by transfection with GFP and iR(A5). Spontaneous NMDAR-dependent activity was detected in HDC (Figure 9F and 9G), as also seen in the eA5-treated neurons. However following EphA5 knockdown by transfection with GFP and iR(A5), neurons lacked spontaneous activity (Figure 9H). Moreover, the effects of HDC on the enhancement of expression of NMDAR subunits and PSD-95 (Figure S4B), and on the enhancement of CREB phosphorylation (Figure S4C, D) were almost completely blocked by nifedipine and H89 (PKA inhibitor), which act upstream from PKA activation in the proposed EphA5-induced CREB phosphorylation pathway in LDC neurons. The effects of HDC on CREB phosphorylation were not blocked by wortmannin, which does not lie on the proposed EphA5-regulated CREB phosphorylation pathway. Furthermore, the effect of HDC on the maturation of spine morphology was almost completely blocked by nifedipine, by H89 and by wortmannin (Figure S4C), in agreement with the observed dependence of EphA5-induced LDC neuron spine maturation on VSCCs, PKA and PI3K/cdc42. These results suggest a similarity between HDC on one hand and LDC neurons in which EphA5 is activated on the other, and support the physiological significance of cell-cell contact for the induction and enhancement of EphA5 signals that induce neuronal maturation.

Recording of naïve membrane potentials showed that neurons in HDC (Figure 9F) or neurons treated with eA5 (Figure 7E), but not control or long-term cultured neurons (Figure 7A, D), were in most cases depolarized above −50 mV. For eA5-treated neurons, the true membrane potentials are actually higher because current injection was applied. Without current injection, eA5-treated neuron membrane potentials were −40 to −30 mV. Depolarization of neurons to membrane potentials above −50 mV in eA5-treated neurons and HDC is significant because it may contribute to activation of VSCC, which is an essential step in our pathway (see Discussion).

## Discussion

### EphA5 signals are involved in the early stages of synaptogenesis

**Figure 10:**
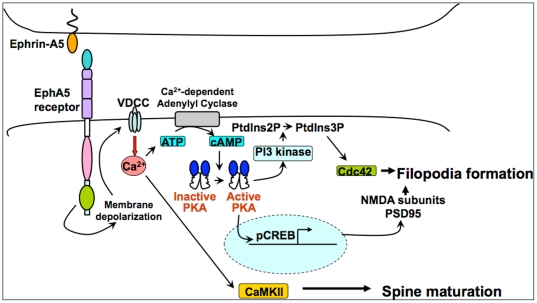
Pathway of EphA5 receptor activation of synaptogenesis and electrical activity. Cell-cell contact activates ephrinA5-EphA5 receptor signaling during early development, prior to the onset of electrical activity. EphA5 receptor signaling induces the formation of synapses and spines with electrically active NMDA receptors. EphA5 may also activate cdc42 through an as-yet-unidentified GEF (not shown). See text for a presentation of the full pathway.

Here we show that early in development, ephrin-A5/EphA5 signals are capable of triggering synaptogenesis, including (1) the expression of functional NMDAR complexes, (2) morphological maturation of spines, (3) activation of the CREB transcriptional factor, and (4) generation of NMDAR-related electrical activity. A common, VSCC-PKA-related pathway regulates these events (Figure 10). Specifically, we show that EphA5 activates voltage sensitive Ca^2+^ channels, possibly through a partial depolarization of membrane potentials. VSCCs elevate cAMP, which activates CREB, and the expression of NMDAR subunits and PSD-95. The activation of PKA stimulates PI3 kinase as well, which activates cdc42 and the genesis of filopodia. Also downstream from VSCC is the activation of CaMKII, whose function is required for the maturation of filopodia to mushroom shaped, mature spines. The consequence of these pathways is the formation of functional NMDAR complexes and the formation and maturation of spines. Notably, AMPARs and their specialized scaffolds are not induced, which is consistent with these being dependent on neuronal activity for their induction and their synaptic trafficking. The roles of ephrinA5 - EphA5 were indicated by the specificity of stimulation by ephrin-A5/Fc, by tyrosine phosphorylation of EphA5 and the specific effects of RNAi knockdown of EphA5. Most significantly, NMDAR EPSCs were absent from hippocampal slices from P6 EphA5 functional knockoutmice but were readily detected in wild type, which confirmed the role of EphA5 indicated by RNAi knockdown. Because these processes were enhanced by cell-cell contact at high cell density, and because stimulation of EphA5 by soluble ephrin-A5 complexes compensated for low-density culture, the ephrin-A5/EphA5 signaling is likely to result from a *trans* interaction that originates from cell-cell contact, which is favored under high cell density growth conditions and is found in hippocampal lamina, rather than *cis* interaction. Also NMDAR receptor subunit subtypes and PSD-95, but not the AMPAR subunits, GluR1 and GluR2, or their scaffolds, GRIP-1 and -2, were enhanced by high cell plating densities. **S**imilar results were obtained for induction of cAMP levels and for spine maturation, all reinforcing a *trans* mechanism. In the latter, eA5 treatment of 6 DIV LDC neurons induced shorter and wider, more mature spines, which required much longer culture (2-3 weeks) in the control.

Eph receptors and their ephrin ligands have a well established role in spine formation and synaptogenesis. Multiple EphB receptors control spine morphology in hippocampus [17]. EphrinB stimulation of EphB2 induces EphB2 binding to the NMDA receptor [14] and activation of kalirin 7 [21] and TIAM [22], GEFs for the small GTPase, Rac, which promotes spine formation and maturation and controls intersectin, a GEF for the small GTPase, cdc42 and cdc42 activation [38]. EphB2 can also stimulate FAK, which induces filopodial transformation into spines via RhoA suppression of cofilin [19], [20]. Furthermore, EphB2 phosphorylates and cluster syndecan-2, which contributes to hippocampal spine formation [39]. EphB2 signaling is required as well for filopodial motility that contributes to synaptogenesis [18].

In contrast to the spine inducing and maturation functions of the EphB receptors, EphA4, which is found on spines and nerve terminals in hippocampus [24], induces spine and dendrite collapse. When EphA4 is stimulated by ephrinA1, it activates Cdk5, which regulates Src phosphorylation of ephexin, which collapses spines and dendrites via activation of RhoA [26]. Ephexin induction by this pathway can also collapse growth cones [40]. The EphA receptors have roles in hippocampal function and memory formation. Inhibition of ephrinA/EphA receptor signaling by soluble EphA5 extracellular domain fragments demonstrated a role of EphA receptor signaling in synaptogenesis and hippocampal connectivity, most likely acting at the postsynaptic side [41], and disrupted memory-based behavioral tasks [42].

EphA5, which has been analyzed in the present report, appears to act at an earlier stage of synaptogenesis than either EphB2 or EphA4 since it can induce the initial expression of NMDA receptors, which in turn bind and regulate EphB2 [16]. Also, unlike EphA4, which induces spine collapse [26], we show here that EphA5 induces spine maturation.

The contributions of ephrin-A5 and EphA5 have been studied by genetic means [43], [44], and a role in synaptogenesis for signals from other ephrins/EphRs at pre-existing synapses has been demonstrated [14], [39]. Late developmental stage establishment and refinement of synapses is closely linked to synapse activity [45]. The current work investigates a less extensively studied part of this program, the early developmental stage, prior to the onset of electrical activity. Indeed, because EphA5 is highly expressed during development of the hippocampus, [31], [32], [33], the pathway studied here may be specific to the early developmental period. Furthermore, because the synaptogenic functions of EphA5 are induced by cell-cell interactions, the dense packing and resulting close proximity of neurons in hippocampal lamina may contribute to early stage synaptogenesis.

The EphB1, EphB2 and EphB3 receptors each make a contribution to spine formation as shown by the progressive reduction of spine upon genetic knockout of one versus two or all three of these genes [17]. While stimulation of the EphB2 receptor by ephrinB2 induced spine formation dependent on the function of kalirin-7 [21] and TIAM [22], induction of spines via stimulation of the EphB1 receptor depended on numb [46]. We have shown that, under high-density culture conditions where EphA5 is active in the absence of exogenous ephrins, spine formation depends strongly on EphA5. The dependence on EphA5 suggests that the level of EphB receptor activity under these HDC culture conditions is not sufficient for spine formation. In agreement, our knockdown of EphB1 or EphB2 under the HDC culture conditions did not suppress spine formation.

### Regulation of NMDAR expression by ephrin/Eph signaling

Although functional NMDAR complex stoichiometry is controversial, the major synaptic NR1/NR2A/NR2B/PSD-95 NMDAR complex induced here by EphA receptor signaling is likely to be physiologic in that it resembles the NR1/NR2A/NR2B complex in cerebral cortex in adult [47]. The pathway of early stage EphA5 induction of the NMDAR-PSD-95 complex involved VSCC, including channels containing the α1D subunit, a subunit that is expressed at this stage [48], but did not involve the NMDAR, a second potential source of Ca^2+^ currents. This mechanism is consistent with EphA5 receptor function prior to NMDAR expression. The induction pathway required activation of PKA. VSCC may activate cAMP synthesis via Ca^2+^/calmodulin-sensitive adenylyl cyclase isoform(s) such as types 1 or 8, which are abundant in brain [29]. This would enable Ca^2+^ fluxes through VSCC to activate PKA in response to EphA5 receptor activation. In more mature neurons, specifically in cerebellar granule cells or hippocampal neurons, cell depolarization by high K^+^, a treatment that reproduces aspects of electrical activity, induced the expression of NR1 and NR2A and repressed the expression of NR2B [49]. This was dependent on Ca^2+^ influx via NMDARs as well as VSCC [30]. Thus mature, electrically active neurons could activate cAMP via the Ca^2+^ conductance of NMDARs and VSCCs, while early stage neurons that lack functional glutamate receptors may utilize VSCC exclusively.

Neither EphA receptor activation nor PKA activation nor cell-cell contact induced expression of AMPAR and their scaffolds. Thus the coordinated expression of the NMDAR and PSD-95 may itself be the developmental switch that induces expression of AMPAR and their interacting proteins. Neurotrophins, including BDNF, regulate AMPAR expression [50] and BDNF and its high affinity receptor, TrkB, are enhanced during development [51], [52]. This suggests that the regulation of expression of glutamate receptors may be divided into an early stage in which ephrin/Eph signals induce NMDAR, and a late stage in which neurotrophins or NMDAR-dependent Ca^2+^ fluxes induce AMPAR and associated proteins.

### Spine formation induced by ephrin/Eph signaling

The formation of mature synapses in pyramidal cells requires filopodial induction and the subsequent maturation of filopodia into spines. We found that HDC and EphA5 each induced the formation of filopodia and the maturation the filopodia into mushroom shaped spines. A similar induction and maturation of filopodia by PKA and PI3K has been reported [53]. CaMKII is also implicated in spine formation [54] and can bind to F-actin and regulate its polymerization [55]. Inhibition of CaMKII in eA5-treated LDC neurons or in HDC led to the formation of filopodia, but not to mature spines. Thus, the activities of PKA and PI3K may induce filopodia, whereas CaMKII activity may facilitate F-actin polymerization, resulting in morphologic maturation and mushroom-shaped spines. PKA activates cdc42 in non-neuronal cells [56] and cdc42 is capable of inducing filopodia [6]. Thus, the intracellular Eph signals that generate filopodia may be transmitted via cdc42 [57]. PKA activity also targets the NMDAR to synapses [58], consistent with the current results.

### Induction of electrical activity by ephrin/Eph signaling

Our work suggests that activation of EphA5 induces functional NMDARs and early stage activity dependent on the NMDAR. Such NMDAR expression could induce electrical activity in developing neurons., even in the absence of AMPAR expression. The activation of NMDARs requires relief of the Mg^2+^ block [5], [7], which may be realized through the membrane depolarization that we observe in eA-treated and HDC neurons (see Results). Later in development, electrical activity would be AMPAR-dependent. Thus, spontaneous activity, which contributes to early synaptogenesis, may involve the NMDAR, whereas use-dependent and experience-driven activity encountered late in development and that is involved in terminal nervous system differentiation and plasticity may involve both the NMDAR and the AMPAR [59].

### Signal transduction following ephrin/Eph activation

We find that in HDC and eA5 treated neurons, but not in control or long-term cultured neurons, membrane potentials are depolarized above −50 mV. Such depolarization could activate VSCC, which in turn could enable a continuous supply of Ca^2+^ into the cytosol. Such a process is in agreement with our finding that VSCC are essential for the early stage synaptogenesis induced by HDC and by ephrin-A5/EphA5. For eA5-treated neurons, the actual membrane potentials are higher than −50 mV because current injection was applied. Without current injection, eA5-treated neuron membrane potentials were −40 to −30 mV, conditions previously reported to activate L-type channels formed with the α1D subunit [60]. It has been shown that in the pyramidal cells of acute hippocampal slices that the membrane potentials are −40 to −50 mV at the neonatal stage, and shift to below −65 mV after P13 [61]. Also, the similarity between membrane potentials in HDC and in neonatal slices supports the conclusion that HDC reflects *in vivo* conditions. This mechanism would also be consistent with dependence of the early development program on the exposure of neurons to eA5 for 6 days for LDC neurons and 4 days for slices. These exposures, which were chosen to mimic exposures by cell contact mechanisms in vivo, may be needed to maintain VSCC activity during the period of early development.

Because of their relatively slow activation kinetics, L-type Ca^2+^ channels respond to sustained or repeated stimulation, rather than to brief depolarization, such as results from a single action potential [62]. Given the immature state of synaptogenesis in hippocampal neurons at the neonatal stage, L-type Ca^2+^ channels in neonatal neurons may utilize a sustained elevation of the membrane potential induced by the ephrin/Eph signal for channel activation. The developmental change in the values of membrane potentials can be explained by a parallel developmental increase in the potassium conductance and by a decrease in the chloride permeability together with a decrease in the intracellular chloride concentration [63], [64], [65], [66]. EphA5 activation might induce membrane depolarization through modulation of these ionic gradients and membrane permeability. Given the extensive colocalization of EphA5 and α1D, Eph signaling in HDC may act on VSCC either directly or indirectly, the latter for example potentially via the release of Ca^2+^ from endoplasmic reticulum by inositol 1,4,5-triphoshate, which activates VSCC.

The EphA5 receptor mechanism of cdc42 activation could involve an as-yet-unidentified GEF, which would be activated by a depolarization-independent mechanism. Indeed, EphA activation of ephexin provides an alternative route for cdc42 activation [26]. Notably, presynaptic Eph receptors at the *drosophila* neuromuscular junction can activate cdc42, which in turn can activate the voltage gated calcium channel, Ca_V_2.1. [67]. Also, RhoA can regulate L-type Ca^2+^ currents in cardiac myocytes [68]. Thus, G-protein dependent pathways could activate the VSCC and could supplement the depolarization-dependent pathway. Activation of a VSCC could in itself provide the partial depolarization that we observe. Although the pathway of VSCC activation is not fully established, the present findings support the involvement of cell contact-dependent ephrin-A5/EphA5 signaling in the mechanism of VSCC activation.

Increases in intracellular Ca^2+^ via VSCC may increase cAMP via Ca^2+^-regulated adenylyl cyclases. Ca^2+^ and cAMP may then work in parallel to activate protein kinases, including PKA and CaMKII [30]. Subsequently, PKA may activate CREB, and CREB may regulate gene expression to stimulate synaptogenesis [69]. In fact, NR1 expression is regulated transcriptionally via PKA, consistent with the involvement of both PKA and phosphorylation of CREB in this up-regulation [70]. PKA may also activate PI3K, shown here to induce spine morphological changes.

### Experimental and clinical relevance of ephrin/Eph signaling

Disruption of neuronal migration at the developmental stage causes specific neuronal disorders [71], including lissencephaly/double cortex syndrome, disorders that are associated with hippocampal cellular dispersion and low cell density [72]. As we have noted, our work suggests that neuronal maturation via the EphA5 signaling pathway depends upon cell contacts that may be provided *in vivo by* the close proximity of neurons the laminar organization of the hippocampus and the cortex. The identification of a cell contact-dependent program that drives the initial stages of hippocampal synaptogenesis may be significant not only for understanding neuronal maturation mechanisms, but also for devising therapies for treatment of neuron migration diseases, and for the preparation of stem cells for injection into hippocampus during restorative stem cell therapy.

## Materials and Methods

The experimental procedures were in accordance with the regulations of the Animal Care Committees of New York University School of Medicine, Osaka University Graduate School of Medicine, and Rutgers University and these institutional review committees specifically approved this study. The approval numbers were: 090403-01 for Animal Care Committee of New York University School of Medicine; and 93-052 for the Animal Care Committee of Rutgers University. The experimental plan submitted by Yukio Akaneya was approved by the Gene Modification Experiments Safety Committee of Osaka University on 9th September, 2003. The approval number is 1882. The procedure for generation of EphA5^lacZ/lacZ^ mice has been described previously [43].

### Nissl staining

Brains for E16 or P6 Sprague Dawley (SD) rat were fixed with 4% paraformaldehyde, followed by addition with 10% sucrose. Two days later, 60–80 µm coronal sections were made using a microtome. After dehydration with ethanol and delipidation with xylene the sections were stained with cresyl violet, followed by treatment of rosin.

### Dissociated Cell Cultures

Hippocampus from E18 SD rat brain was dissected and dissociated, followed by plating on poly-*L*-lysine-coated dishes at 6×10^3^,18×10^3^, 54×10^3^, or 162×10^3^ cells/cm^2^ for eventual Western analysis, or at 18×10^3^ or 162×10^3^ cells/cm^2^ for IP, immunostaining or cAMP assay. The cells were cultured at 37°C for 3 hr, and the plating medium was exchanged for the Neurobasal media containing B27, 0.5 mM glutamine. Throughout the experimental period (6 DIV), proliferation of non-neuronal cells such as glial and endothelial cells was suppressed to less than 6% of total cells.

### Organotypic Slice Cultures

Hippocampus from P0 SD rat brain was dissected followed by slicing with a McIlwain type tissue chopper (Stoelting). Then the slices were plated on Millicell culture plate inserts (0.4 µm pore; Millipore) in 6 well-plates containing medium consisting of 50% MEM (Gibco), 25% Hank's balances salt solution (Gibco), 25% horse serum, 6.5 g/l glucose and the above-mentioned antibiotics. These slices were incubated at 33°C until analysis.

### Western blot analysis

Harvested cells were solubilized by rocking incubation with 25 mM HEPES, 150 mM NaCl, 1 mM EDTA and 1% Triton-X100 for 30 min at 4°C. Lysates were analyzed by SDS-PAGE and Western blotting on nitrocellulose filters. The primary antibodies used for protein visualization, their dilution and the companies from which antibodies were purchased are the following: rabbit polyclonal anti- (rpa-)ephrin-A5, 1: 250–500 (Santa Cruz); rpa-EphA5, 1: 250–500 (Santa Cruz); mouse monoclonal anti-(mma-)phospho-tyrosine, 1; 500 (Cell Signaling); mma-NR1, 1∶500 (PharMingen); goat polyclonal anti-NR2A, 1∶500 (Santa Cruz); mma-NR2B, 1∶250 (Transduction Lab.); rpa-GluR1, 1∶500 (Chemicon); rpa-GluR2, 1∶500 (Chemicon); mma-PSD-95 (28/43), 1: 3,000 (Upstate Biotech); mma-α-tubulin, 1: 15,000 (Sigma), rpa-GRIP [4], 1: 500; rpa-ABP, 1: 500 [4], rpa-α1D, 1∶500 (Alomone); mma-CREB, 1∶500 (Cell Signaling), rpa-pCREB, 1∶500 (Cell Signaling). The mean of intensities of selected areas and the areas of these images were calculated using NIH image software.

### Immunostaining

Cells on glass coverslips, and the tissue or organotypic culture slices were fixed with 4% paraformaldehyde for 10 min, at room temperature and overnight at 4°C, respectively, followed by permeabilization with 0.2% Triton-X100 for 5 min, except with NR1 staining, for which cells were fixed with methanol for 15 min at −20°C. Then cells were incubated with 10% goat serum in PBS, followed by incubation with primary antibodies overnight at 4°C. The dilution of primary antibodies and the companies from which antibodies were purchased are the following: mma-NSE, 1∶30 (Santa Cruz); goat-polyclonal DCX antibody, 1∶50 (Chemicon); nmma-NR1, 1∶100 (PharMingen); rpa-NR2A, 1∶200 (Molecular Probe); mma-NR2B, 1∶100 (Transduction Lab.); rpa-NR2B, 1∶200 (Molecular Probe); mma-synaptophysin; 1∶100 (Sigma); rpa-synaptophysin, 1∶3,000 (Zymed); mma-PSD-95 (28/43), 1∶100 (Upstate Biotech); chicken polyclonal anti-GFP, 1∶2,000 (Chemicon); mma-EphA5,1∶100 (R & D Systems); rpa-ephrin-A5, 1: 100 (Santa Cruz); rpa-α1D, 1∶100 (Alomone); rpa-phospho-CREB, 1∶100 (Cell Signaling). After washing with PBS, cells were incubated with FITC- and Texas Red-conjugated secondary antibodies or with FITC-, Texas Red- and Cy5-cojugated antibodies for 1 hr at room temperature. Immunofluorescence signals were imaged through a confocal microscope (Nikon PCM 2000) and SIMPLE 32 software. For GFP expression experiments, we used a two-photon laser-scanning system (Radiance 2000MP; Bio-Rad, Hertfordshire, UK) coupled with a mode-locked Ti:sapphire laser (950 nm; Spectra-Physics, Mountain View, California) pumped with a 10 W solid-state source (Millenia PRO 10sJ 110; Spectra-Physics). The scanhead was attached to an upright microscope (ECLIPSE E600FN; Nikon, Tokyo, Japan). Excitation light was focused using a 60× water-immersion objective (CFI Fluor; 1.0 numerical aperture; Nikon). The point-spread function of the focal volume at 950 nm was estimated using 0.1-µm-diameter fluorescent beads as 0.45 µm (full-width at half-maximum) laterally and 1.8 µm axially. We obtained images along the *z*-axis were stacked with each *z*-axis section separated by 0.5 µm and the fluorescence values for each pixel were summed. Lengths and widths of spines were measured with MetaMorph software.

### Membrane fraction purification and immunoprecipitation

Membrane fractions were isolated as described previously [73]. Equal amounts of lysate protein were immunoprecipitated with mma-NR1 (PharMingen), goat polyclonal anti-NR2A (Santa Cruz), mma-PSD-95 (28/43) (Upstate Biotech), rpa-EphA5 (Santa Cruz) or rpa-ephrin-A5 (Santa Cruz) antibody overnight at 4°C, and then added with protein A-agarose or protein G-agarose, followed by incubation for 3 hr at 4 °C. After washing, samples were boiled at 100°C for 5 min. Supernatants were used for Western blotting. Parenthesis below ‘Blot’ indicates probing of the same membrane in Figure 6A–D.

### Analysis for cdc42 activity

Cdc42 activity was measured with the lysates prepared as described above and analyzed according to the company's instruction (EZ-Detect Cdc42 activation kit, Pierce).

### Preparation for siRNA

Targets for each rat EphA5, EphB1 or EphB2 siRNA were designed with reference to the NCBI library, and the uniqueness of sequence was ascertained with the NCBI nucleotide BLAST program. Oligonucleotide templates for siRNAs preparation were designed as follows;

[targeting siRNAs]

iR(A5)-antisense:AACGAAGTGAATTTATTGGATCCTGTCTC

iR(A5)-sense:AAATCCAATAAATTCACTTCGCCTGTCTC

iR(B1)-antisense:AAGTACCTATCTGAGATGAATCCTGTCTC

iR(B1)-sense:AAATTCATCTCAGATAGGTACCCTGTCTC

iR(B2)-antisense: AACGGCTGAGCTGGGCTGGATCCTGTCTC

iR(B2)-sense:AAATCCAGCCCAGCACAGCCGTTCCTGTCTC

[control siRNAs]

iR(A5′)-antisense:AATAGTAGCGAAGTGTTATATCCTGTCTC

iR(A5′)-sense:AAATATAACACTTCGCTACTACCTGTCTC

iR(B1′)-antisense:AAGTTATCAATAGCTGACAGTCCTGTCTC

iR(B1′)-sense:AAACTGTCAGCTATTGATAACCCTGTCTC

iR(B2′)-antisense: AACGACCGTGGGGTGAGTTCGCCTGTCTC

iR(B2′)-sense:AACGAACTCACCCCACGGTCGCCTGTCTC

The underlined sequences indicate the region complementary to the T7 promoter primer. The ratios of bases contained in the control siRNA were the same as those of the corresponding siRNA. These sense and antisense oligonucleotides were annealed and then filled in with Klenow DNA polymerase according to manufacture's instructions (Silencer siRNA construction kit, Ambion). These double strand oligonucleotide templates were transcribed with T7 RNA polymerase and hybridized. After RNase digestion, siRNAs were used for transfection.

### Transfection of GFP plasmids and siRNA

For lipofection, using lipofectamine 2000 (Invitrogen), pGFP were transfected into hippocampal neurons 2 or 3 days before morphological observation under the indicated conditions, followed by taking images of living neurons using a two-photon microscope. For siRNA transfection, pGFP and siRNAs for EphA5, EphB1, or EphB2 siRNA were co-transfected into hippocampal neurons of 3 DIV at 162×10^3^ cells/cm^2^, and after 3 or 4 DIV images of living neurons were taken through a two-photon microscope.

For transfections by gene gun, gold particles coated with pGFP and the siRNAs were prepared as follows; 25 µg pGFP and 5 µg siRNA were added to 0.05 M spermidine (Sigma) and 1.0 µm gold particles (Bio-Rad) suspended in the 100 µl of RNase-free water. Then 100 µl of 1 M CaCl_2_ was added into this suspension. This gold/DNA and siRNA/spermidine complex was suspended in 5 ml of 99.5% ethanol, followed by drawing into a silicon-coated tube. This tube was placed in a tubing prep-station (Bio-rad). Then, ethanol was removed slowly. After drying, the tube was cut for use in a cartridge holder of a Helios gene gun (Bio-rad). For transfection, slice cultures at 1 DIV were shot by a gene gun, followed by an additional 3 DIV of cultivation.

### cAMP assay

Hippocampal neurons were cultured for 6 DIV in LDC or HDC with or without APV (50 µM) or nifedipine (20 µM). After harvesting, cAMP in lysates was assayed with a cAMP EIA kit (Cayman) according to the manufacture's instructions.

### Electrophysiology

Spontaneous responses were recorded through glass pipettes (4–6 MΩ resistance) to patch pyramidal cell-like neurons cultured for 6–7 or 16–18 DIV in the current clamp mode. These neurons were visually identified using an upright microscope with Nomarski optics (Axioscope FS, Zeiss, Germany). Recording pipettes were filled with a solution containing (in mM): potassium gluconate 110, KCl 10, HEPES 10, EGTA 0.5, MgATP 5, Na_2_GTP 1, and were adjusted to pH 7.4 by KOH. The osmolarity of the solution was 240–245 mOsm. The input and series resistances were monitored with −0.1 nA current or −10 mV voltage steps. The series resistance was <30 MΩ. The input resistance was 300 to 800 MΩ. Responses were recorded with a patch-clamp amplifier (Axoclamp 2B, Axon Instruments, Foster City, CA) filtered at 2 kHz, digitized at a rate of 10 kHz and fed into an IBM-PC clone computer for analysis.

Transverse hippocampal slices (300 µm) were made from 6-days old mice with a vibratome (Campden Instruments) and maintained at room temperature for 90 min in brain slice keeper (Scientific Systems Design Inc.) before transferring to a recording chamber. Experiments were conducted using whole cell patch clamp recordings from CA1 pyramidal cells by stimulating the Schaffer collaterals. NMDA EPSCs were recorded at 31°C in presence of NBQX (10 µM) and bicuculline (10 µM) by holding the neuron at +40 mV. The extracellular solution contained 118 mM NaCl, 2.5 mM KCl, 10 mM glucose, 1 mM NaH_2_PO_4_, 3 mM CaCl_2_, 2 mM MgCl_2,_ and 25 mM NaHCO_3_ (osmolarity adjusted to 325 mOsm and aerated by 95% O_2_/5% CO_2_ (pH 7.4). The electrode solution contained 145 mM CsCl, 10 mM HEPES, 0.5 mM EGTA, 5 mM QX-314, and 2 mM Mg^2+^-ATP (Osmolarity is adjusted to 290 mOsm with sucrose, and pH is adjusted to 7.4 with CsOH.). The amplitudes of NMDA EPSCS were plotted against stimulation intensities (0.1–0.9 mA).

## Supporting Information

Figure S1 Treatment with eA5 or eB1 does not alter EphA5 levels. (A) Western blotting of LDC and slice cultures treated with eA5 or eB1 and of hippocampal tissues from wild and EphA5-transgenic mice (EphA5^lacZ/lacZ^). Panels below the blotting of EphA5 and ephrin-A5 show representative blots of the noted protein and α-tubulin. n = 5, 4 and 4, for LDC, slices and *in vivo*, respectively. (B) Quantitation of (A).
(0.34 MB TIF)

Figure S2 Control Eph receptor siRNA knockdowns. (A and B) The scrambled control siRNAs, iR(A5′), iR(B1′) and iR(B2′) had no significant knockdown effect on the noted Eph receptors. (C) Neither the heterologous experimental RNAis, iR(B1) and iR(B2), nor the heterologous control RNAis, iR(B1′) and iR(B2′) showed significant knockdown of EphA5.
(8.67 MB TIF)

Figure S3 Transcriptional regulation of NR1 by 8CPT. Treatment of LDC neurons with 8CPT increased NR1 transcripts approximately eightfold relative to control, suggesting that the regulation by PKA was transcriptional.
(0.19 MB TIF)

Figure S4 Colocalization and regulation of NMDAR subunits and PSD95 with synaptophysin induced by HDC. (A) NR1, NR2A, NR2B, PSD-95 and synaptophysin colocalize in HDC neurons. (B) HDC enhancement of expression of NMDAR subunits and PSD-95 is blocked by nifedipine and H89 but not wortmannin. (C) HDC enhancement of spine morphology was blocked by blocked by nifedipine, by H89 and by wortmannin. (D) CREB phosphorylation was blocked by nifedipine and H89 (PKA inhibitor) but not by wortmannin. See Text.
(1.10 MB TIF)
