# Supplementary figures and images for: Ephrin-A5 and EphA5 Interaction Induces Synaptogenesis during Early Hippocampal Development
Source: PLoS One. 2010 Aug 31;5(8):e12486. doi: 10.1371/journal.pone.0012486 (PMC2930854; doi:10.1371/journal.pone.0012486)

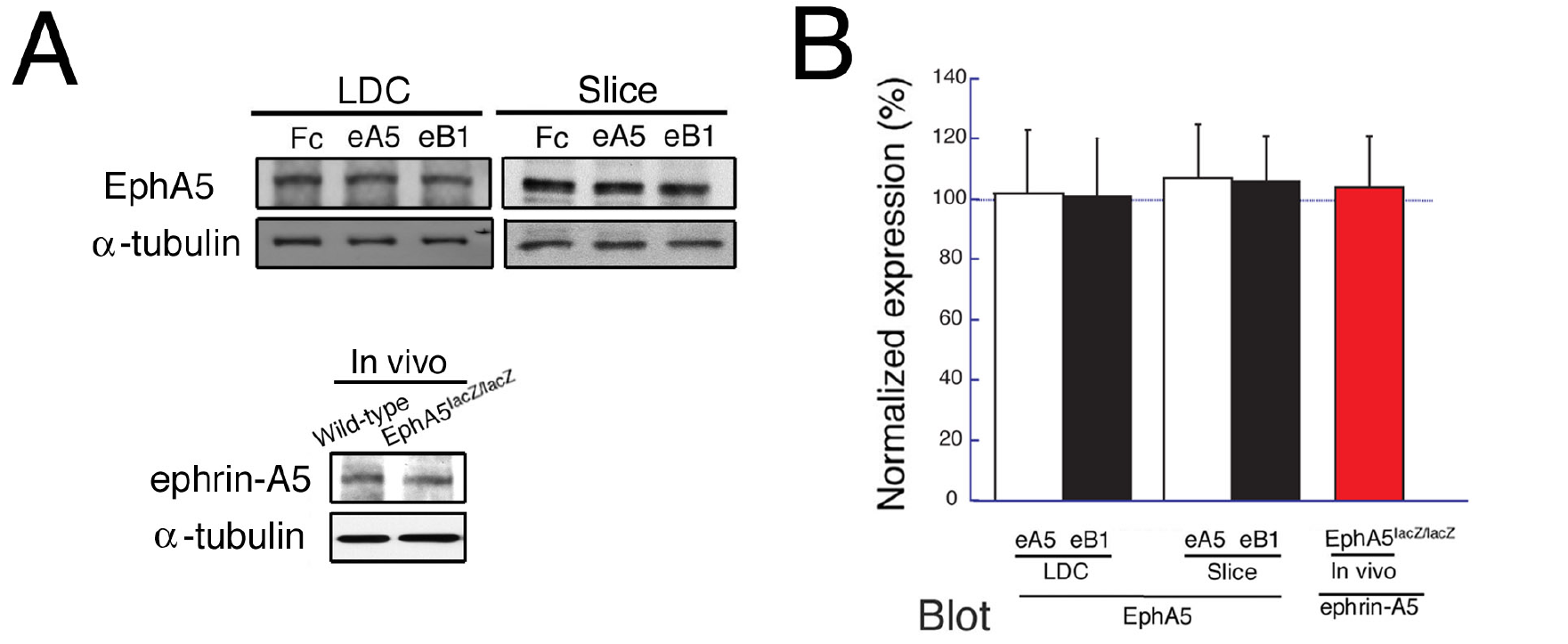

Supplement: Figure S1 — Treatment with eA5 or eB1 does not alter EphA5 levels. (A) Western blotting of LDC and slice cultures treated with eA5 or eB1 and of hippocampal tissues from wild and EphA5-transgenic mice (EphA5lacZ/lacZ). Panels below the blotting of EphA5 and ephrin-A5 show representative blots of the noted protein and α-tubulin. n = 5, 4 and 4, for LDC, slices and in vivo, respectively. (B) Quantitation of (A). (0.34 MB TIF) [file pone.0012486.s001.tif]

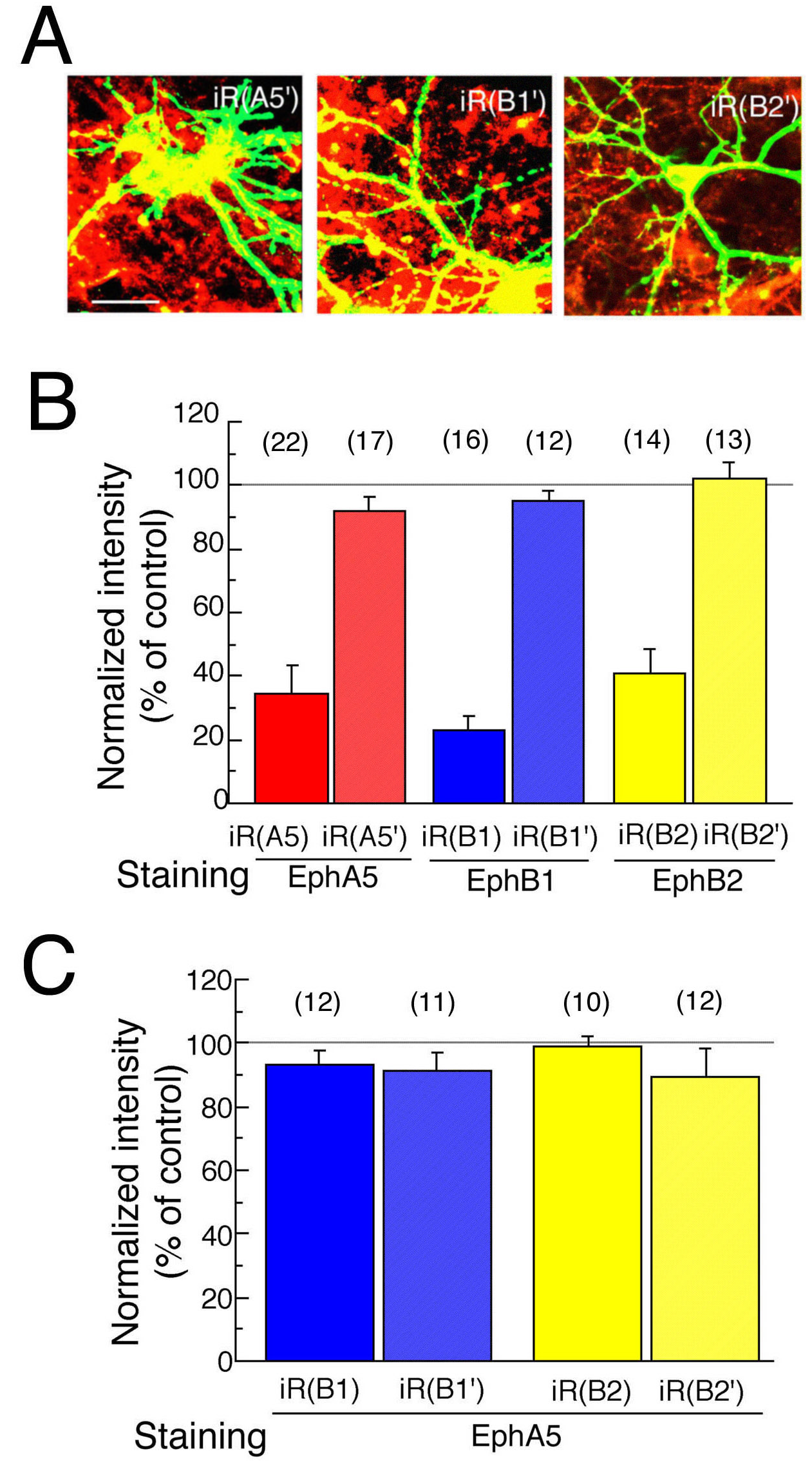

Supplement: Figure S2 — Control Eph receptor siRNA knockdowns. (A and B) The scrambled control siRNAs, iR(A5′), iR(B1′) and iR(B2′) had no significant knockdown effect on the noted Eph receptors. (C) Neither the heterologous experimental RNAis, iR(B1) and iR(B2), nor the heterologous control RNAis, iR(B1′) and iR(B2′) showed significant knockdown of EphA5. (8.67 MB TIF) [file pone.0012486.s002.tif]

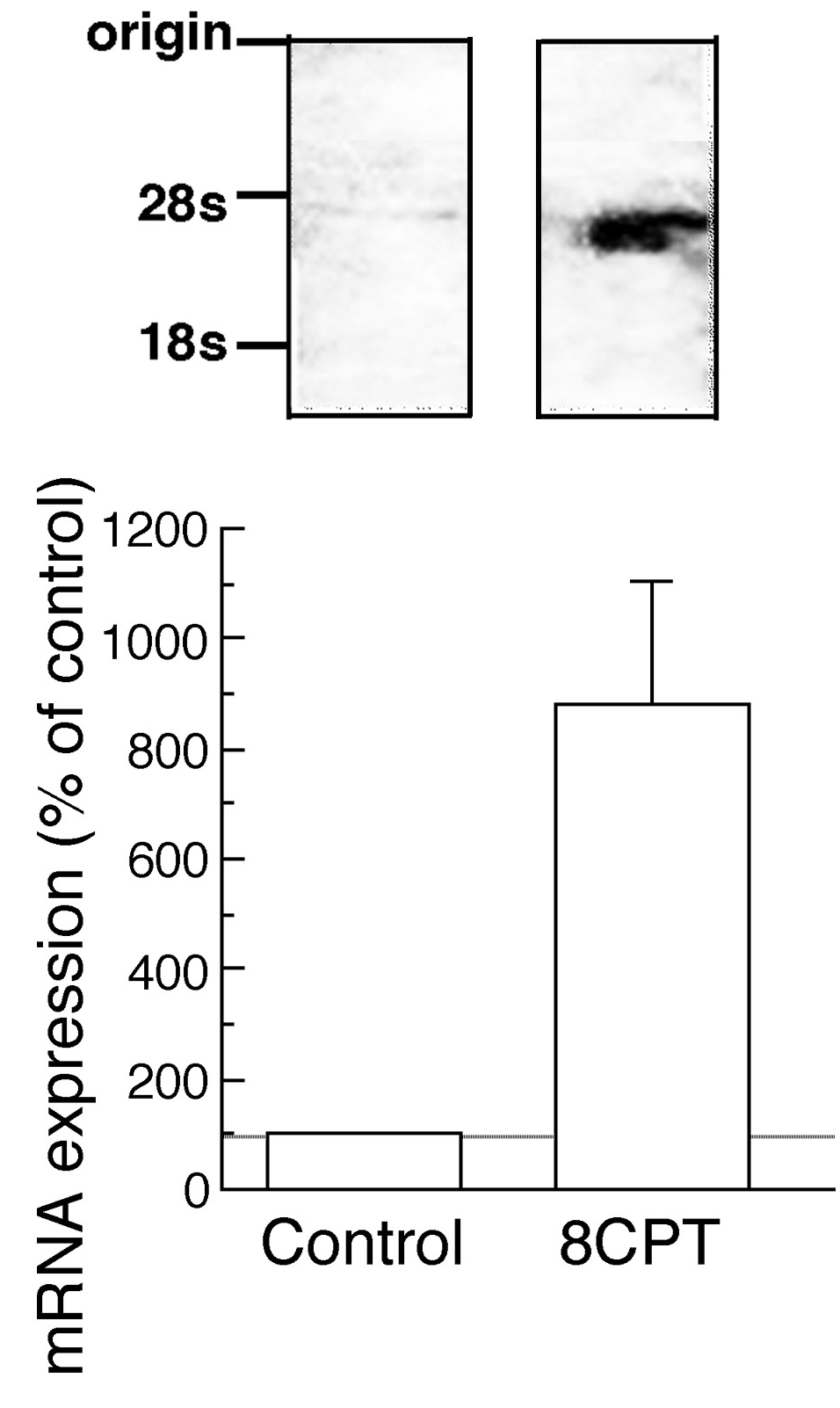

Supplement: Figure S3 — Transcriptional regulation of NR1 by 8CPT. Treatment of LDC neurons with 8CPT increased NR1 transcripts approximately eightfold relative to control, suggesting that the regulation by PKA was transcriptional. (0.19 MB TIF) [file pone.0012486.s003.tif]

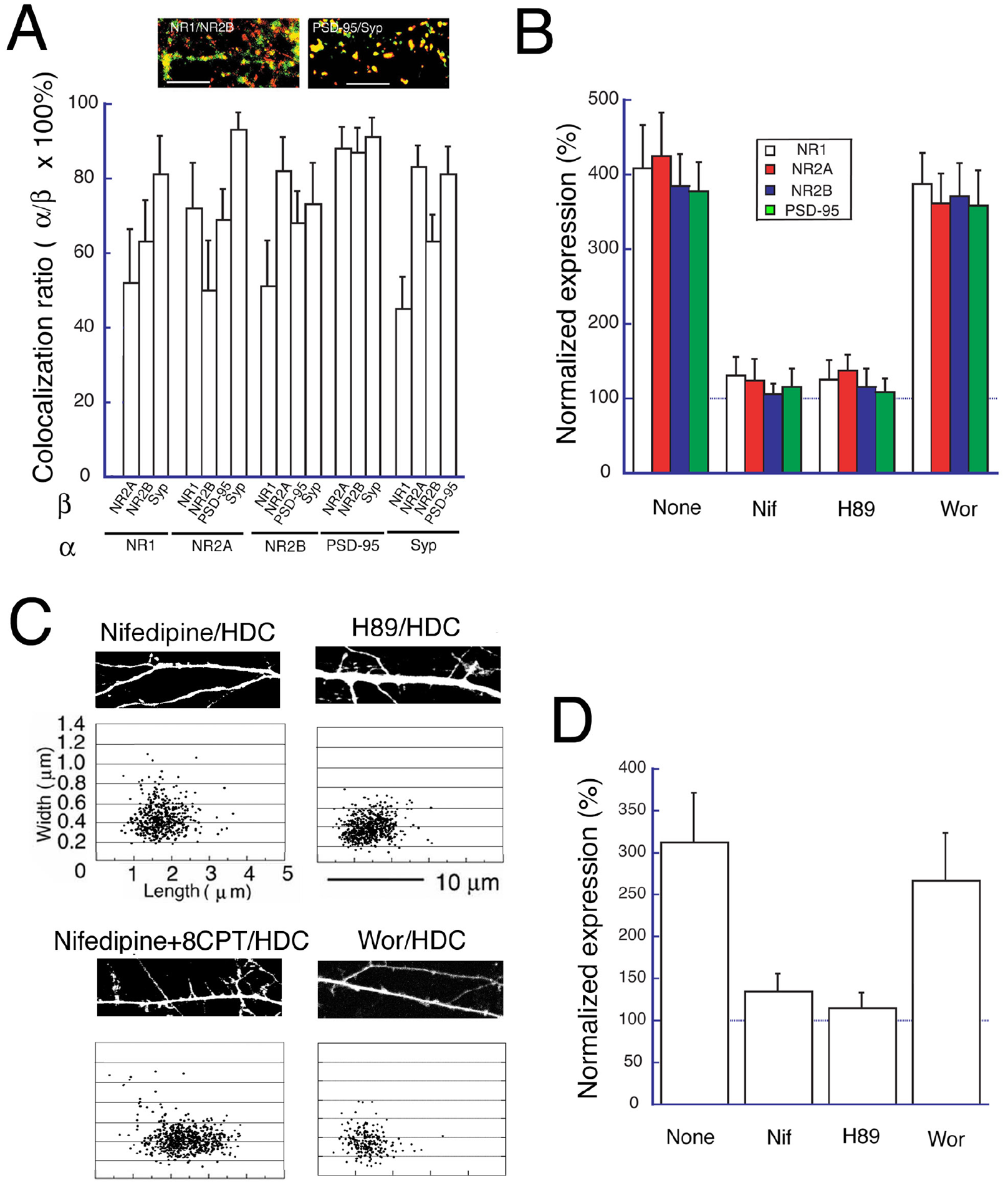

Supplement: Figure S4 — Colocalization and regulation of NMDAR subunits and PSD95 with synaptophysin induced by HDC. (A) NR1, NR2A, NR2B, PSD-95 and synaptophysin colocalize in HDC neurons. (B) HDC enhancement of expression of NMDAR subunits and PSD-95 is blocked by nifedipine and H89 but not wortmannin. (C) HDC enhancement of spine morphology was blocked by blocked by nifedipine, by H89 and by wortmannin. (D) CREB phosphorylation was blocked by nifedipine and H89 (PKA inhibitor) but not by wortmannin. See Text. (1.10 MB TIF) [file pone.0012486.s004.tif]
